# Multidimensional computational study to understand non-coding RNA interactions in breast cancer metastasis

**DOI:** 10.1038/s41598-023-42904-6

**Published:** 2023-09-22

**Authors:** Sohini Chakraborty, Satarupa Banerjee

**Affiliations:** Department of Biotechnology, School of Biosciences and Technology, Vellore Institute of Technology, Vellore, 632014 Tamil Nadu India

**Keywords:** Cancer, Systems biology

## Abstract

Metastasis is a major breast cancer hallmark due to which tumor cells tend to relocate to regional or distant organs from their organ of origin. This study is aimed to decipher the interaction among 113 differentially expressed genes, interacting non-coding RNAs and drugs (614 miRNAs, 220 lncRNAs and 3241 interacting drugs) associated with metastasis in breast cancer. For an extensive understanding of genetic interactions in the diseased state, a backbone gene co-expression network was constructed. Further, the mRNA–miRNA–lncRNA–drug interaction network was constructed to identify the top hub RNAs, significant cliques and topological parameters associated with differentially expressed genes. Then, the mRNAs from the top two subnetworks constructed are considered for transcription factor (TF) analysis. 39 interacting miRNAs and 1641 corresponding TFs for the eight mRNAs from the subnetworks are also utilized to construct an mRNA–miRNA–TF interaction network. TF analysis revealed two TFs (EST1 and SP1) from the cliques to be significant. TCGA expression analysis of miRNAs and lncRNAs as well as subclass-based and promoter methylation-based expression, oncoprint and survival analysis of the mRNAs are also done. Finally, functional enrichment of mRNAs is also performed. Significant cliques identified in the study can be utilized for identification of newer therapeutic interventions for breast cancer. This work will also help to gain a deeper insight into the complicated molecular intricacies to reveal the potential biomarkers involved with breast cancer progression in future.

## Introduction

Research shows that breast cancer (BC) morbidity has been on the rise^1^. BC cell motility is one of the key traits of individual tumor cells. The primary clinical cause of BC morbidity accounts to metastasis^2,3^. Metastasis is the migration of tumor cells from the site of origin to different locations of the body resulting in new tumor colonies that result in deaths^4^. The main sites of BC metastasis include the lungs, liver, bone and brain^5^. The current therapeutic strategies to combat BC are surgery, chemotherapy and radiation therapy^6^. Tumor metastasis is associated with various types of RNAs and compounds that play a role in deregulating different BC signalling cascades to lead to BC progression. Before metastasis, the primary tumor cells secrete cytokines and extracellular vesicles that modulate the pre-metastatic niche formation.

The messenger RNA (mRNA)-microRNA (miRNA)-long noncoding RNA (lncRNA) axis is significantly associated with tumor pathogenesis such tumor stage, rates of progression and metastasis making them potential candidates of clinical outcome or progression prediction including survival and severity of BC. In this study we deal with two categories of noncoding RNAs (miRNAs and lncRNAs) interacting with the differentially expressed metastatic mRNAs.

The miRNAs are endogenous single-stranded noncoding RNAs (ncRNA) having 19–23 nucleotides (nt) that are key regulators in cell development and division^7^. The miRNAs also regulate key signalling pathways and mRNA translation is inhibited or enhanced by posttranscriptional degradation modulating target gene expression and indirectly control tumor cell growth and apoptosis, triggers angiogenesis, and/or controls the cell cycle processes. The lncRNAs play an important role in evolution and progression of BC^8,9^. These noncoding transcripts can affect to different cancer hallmarks like angiogenesis, epithelial-to-mesenchymal transition (EMT), apoptotic cascades, tissue invasion and metastasis to name a few. For instance, NEAT1 promotes progression of BC by modulating CCND1 gene expression^10^, while LINC01355 lncRNA suppresses BC progression via FOXO3-mediated inhibition of CCND1^11^.

DNA methylation is frequently linked to differential gene expression, and until recently, the general consensus was that higher levels of cytosine methylation around and inside genes are associated with regulation of gene expression^12^. Calculating ratios for gene expression across samples to obtain the fold-change (FC) signifies the factor of change in expression between groups. Such genes are called differentially expressed genes (DEGs) which are expressed differently across different types of samples. They exhibit statistically significant variable behavior across samples constructed after marginalizing only those genes that truly demonstrate a difference. Limma (Linear Models for Microarray Data) t-statistical algorithm is generally used to identify DEGs for feature selection. The ground-level idea behind limma is modelling of expression levels of each gene as a linear combination of experimental factors and covariates. It studies gene expression microarray data, with a focus on the evaluation of differential expression and the application of linear models for the analysis of specified experiments. The model coefficients are estimated by maximum likelihood estimation (MLE) alongside hypothesis testing performed on estimated coefficients to determine DEGs across experimental condition with associated significant *p* values and false discovery rates (FDRs). The algorithm initially normalizes the microarray data to denoise and avoid bias of datasets. Also, Benjamini–Hochberg (B–H) correction is employed for reduction of FDR^13,14^. The final computationally exhaustive list of DEGs that has undergone these algorithms and corrections is obtained and are considered for the study.

Already existing drugs including chemotherapeutic agents not only have side effects, but also with prolonged usage of such drugs the BC cells become resistant to the effects of the drug. The only way to minimize side effects and avoid chemoresistance is dosage modifications or modulations of the chemotherapeutic drugs. Chemotherapeutic agents have shown directly or indirectly cause cytotoxicity resulting in tumor regression even after dosage modulations. Moreover, only a handful number of drugs target tumor tissues. Increasing research reveals that BC development and recurrence is not only associated with physiological aspects of the body but also have a direct impact on psychological and social life^15^. It is the need of the hour to explore drugs targeted to treat BC cells that can potentially work without effecting the non-cancerous native cells of the body, thereby mitigating the side effects of existing treatment options with newer therapeutic options.

Hence, in this study, the detection of significant cliques, biomarkers and drugs derived from interaction networks of mRNA–miRNA–lncRNA–drug interactions, followed by their expression, survival and oncoprint profiles can be useful and critical for an enhanced understanding of the BC progression, which may lead to identification of newer therapeutic strategies as well.

## Methodology

### Retrieval of significant mRNAs

The mRNAs associated with BC metastasis are retrieved from the HCMDB database (https://hcmdb.i-sanger.com/)^16^. This integrated database supports wide range of expression data compiled from Gene Expression Omnibus (GEO) (https://www.ncbi.nlm.nih.gov/geo/) as well as The Cancer Genome Atlas (TCGA) datasets pertaining to cancer metastasis. Moreover, the shared genes among the four datasets and Human Cancer Metastasis Database (HCMDB) were explored for distinguishing metastatic target genes of BC using InteractiVenn tool (http://www.interactivenn.net/)^17^. These metastatic mRNAs are then compared to differentially expressed genes (DEGs) in BC obtained from the GEPIA database (http://gepia.cancer-pku.cn/)^18^ to get a final list of 113 differentially expressed metastatic mRNAs involved in BC. GEPIA (Gene Expression Profiling Interactive Analysis) aids in time-efficient and customized functions of genes including the differential expression, correlation status, survival plots, gene similarity indices etc. encompassing the gene set from TCGA (https://www.cancer.gov/ccg/research/genome-sequencing/tcga)^19^ and GTEx datasets (https://www.genome.gov/Funded-Programs-Projects/Genotype-Tissue-Expression-Project)^20^.

### Retrieval of non-coding RNAs

In this study, we deal with two different types of ncRNAs:miRNAs and lncRNAs. The common miRNAs interacting with the DEGs are retrieved from ENCORI (https://starbase.sysu.edu.cn/)^21^, miRTarBase (https://mirtarbase.cuhk.edu.cn/~miRTarBase/miRTarBase_2022/php/search.php)^22^ and TargetScan (http://www.targetscan.org/vert_80/). ENCORI database is composed of experimentally identified interaction networks of RNA–RNA and protein–RNA form 108 CLIP-Seq datasets generated from 37 independent studies. miRTarBase datasets exclusively deals with miRNAs along with high-throughput corroborative miRNA-target interactions. The contemporary miRNA-mRNA interactions are specifically supported from CLIP-Seq verified data. TargetScan gathers anticipated targets of miRNAs by exploring the conserved 8-, 7- and 6-mer sites that complement the seed region of miRNA. In case of common lncRNAs interacting with the DEGs two databases (TarBase v8: https://dianalab.e-ce.uth.gr/html/diana/web/index.php?r=tarbasev8/index^21^ and lncBase v3 https://diana.e-ce.uth.gr/lncbasev3)^23^ are utilised. ENCORI is also used to obtain the miRNAs and lncRNAs that interact with each other.

### Retrieval of interacting drug list

The drugs interacting with mRNAs are retrieved from the Comparative Toxicogenomics Database (https://ctdbase.org/)^24^. CTD comprises of manually curated mRNA–drug interactions that affects various biological pathways underlying various diseases such as cancer. For information regarding drugs interacting with miRNAs, Sm2miR (http://www.jianglab.cn/SM2miR/)^25^ is utilised. Sm2miR database is an inclusive database consisting of information of the role of drugs and various small molecules affecting miRNA expression influencing miRNA-associated therapeutics. The lncRNA–drug interaction data is retrieved from the D-lnc database (http://www.jianglab.cn/D-lnc/)^26^.

### Generation of mRNA–miRNA–lncRNA–drug interaction network followed by hub RNA, module identification and TF analysis

In order to understand the interaction in the complex linkage of miRNA–mRNAs–lncRNA–drugs, the cytoscape v3.9.0^27^ software was utilized effectively. It is a freely accessible platform to perform complex cluster- based networks for multiple bioentities approach. The software enables visualization of complex networks comprising of multiple bioentities. From the ‘Tool” dropdown menu, ‘Merge” attribute has been used utilized to merge the small networks formed namely, mRNA–mRNA, mRNA–lncRNA, mRNA–drugs, miRNA–drugs, lncRNA–drugs and miRNA–lncRNA. Consequently, the Maximal Clique Centrality (MCC) ranking method of the ‘cytohubba’ plugin has been utilized for extracting the hub mRNAs from interaction network. Additionally, the ‘MClique’ plugin was employed to obtain cliques. The MCODE plugin is employed on the interaction network to obtain the top two subnetworks (based on MCODE Score of 5.438 and 5.417) to carry out transcription factor (TF) analysis, respectively. The top BC carcinogenic conditions in which the highly expressed hub genes (cut-off degree of 30 for significant genes) are obtained from the topological parameters. For the mRNAs from the top two subnetworks from MCODE results, interacting miRNAs are searched from the ENCORI database. Once the mRNAs and miRNAs are retrieved, the interacting TFs for mRNAs and miRNAs are retrieved from the TRRUST (https://www.grnpedia.org/trrust/)^28^ and TransmiR (https://www.cuilab.cn/transmir)^29^ databases, respectively. To retrieve the hub TFs, the subnetworks are first merged and then MClique plugin is employed on the interaction network involving mRNAs, miRNAs and TFs. ChA3 (https://maayanlab.cloud/chea3/)^30^ TF analysis was done for all the TFs obtained from TRRUST and TransmiR to validate the hub TFs revealed in the Clique identification in MClique of cytoscape. To validate the significance of the TFs, CheA3 TF analysis of the TFs was done.

### Status of various correlated gene regulation in diseased state

In TCSBN, the gene co-expression network for the 20 hub RNAs is built under the category of cancer tissue utilising the Breast (BRCA) dataset. Based on the hub RNAs retrieved from the interaction network, a backbone gene co-expression network is built using the Tissue/Cancer-Specific Biological Networks (https://inetmodels.com/)^31^ database. Among the top 20 hub RNAs, 11 were mRNAs. The adjusted *p* value is set to 0.05, the node limit is 25, correlation is set to both (positive and negative).

### Expression, survival and oncoprint analyses of the RNAs

Following the retrieval of hub RNAs, the expression analysis for mRNAs and miRNAs is done using UALCAN (The University of ALabama at Birmingham CANcer). The UALCAN webtool (http://ualcan.path.uab.edu/cgi-bin/ualcan-res.pl)^32^ generally comprises of omics data related to cancer and provides efficient expression profiles for genes corresponding to protein-coding and non-coding RNAs (ncRNAs). The gold-standard metastatic dataset of TCGA corresponding to ‘BRCA: breast invasive carcinoma’ dataset is exploited effectively to retrieve the expression pattern of the hub RNAs. For the selections pertaining to sample types, BC subclasses and their subclass-associated DNA-methylation status, genes with *p* value < 0.05 were checked for their significant expression in BC. Subclass-based promoter methylation status is generally an epigenetic event in the initial phases of tumorigenesis and therefore has prognostic cancer biomarker potential^33^. In order for normal regulation of the genes, DNA methylation as well as structure of the chromatin play significant roles. Hence, DNA methylation status was also checked using UALCAN tool. The beta-value along the ordinate-axis of the methylation plots range from 0 (unmethylated) to 1 (full methylated mRNAs). Hypermethylation ranges from beta value of 0.7–0.5 while the 0.3–0.25 range of beta value indicates hypomethylation.

Further, survival analysis of mRNAs was performed using Oncolnc (http://www.oncolnc.org/)^34^ that provides an interactive platform correlating the survival data of cancer patients obtained from TCGA with mRNA, miRNA and lncRNA expressions. The patients’ tumor samples were divided into high- and low-expression groups (n = 503) which were analyzed by log-rank test and statistical significance of the selected markers was confirmed with *p* value < 0.05. To validate the most significant survival biomarkers PrognoScan (http://dna00.bio.kyutech.ac.jp/PrognoScan/)^35^ and Fp tool (http://dcv.uhnres.utoronto.ca/FPCLASS)^36^ scores were compared to the UALCAN *p* values for individual DEGs. The robust platform provided by PrognoScan makes it possible to assess prospective tumor markers and treatment targets, which would speed up the study of cancer. Finding the genes that are co-expressed with hub genes can be done using the in-silico method known as the Fp tool, which predicts high-confidence protein–protein interactions. Based on the total scores, the co-expressed genes were determined.

In order to achieve vital information on the genetic alteration of the DEGs in individual hallmarks, oncoprint analysis was performed with the help of cBioPortal server (https://www.cbioportal.org/)^37^. The Oncoprint technique is particularly helpful for finding trends like co-occurrence and mutual exclusivity as well as for visualizing changes across a group of instances. In the end, two genes that do not co-occur in the same patient may be in the same pathway. Finding these genes makes it possible to develop artificially deadly treatments. It shows discrete gene values for all data types, including data with continuous values like mRNA expression (i.e., whether a gene is altered or not based on a predetermined threshold. This integrative platform provides high quality genetic profiles relative to the various alterations at molecular level. Samples across the TCGA dataset (Cell, 2015) was explored to see genetically altered mRNAs. This database reveals different chromosomal mutations with their chromosomal position and changes in base pair. In addition to the cBioPortal default layout, there are supplementary bar plots on either side of the heatmap that display the number of various modifications for each sample and each gene.

In this study we have incorporated this data in the form of a Circos plot using the web-based Circos tool (http://mkweb.bcgsc.ca/tableviewer/)^38^.

### Enrichment analysis of the DEGs

EnrichR was used to conduct the functional enrichment analysis of the DEGs. This web-based tool (https://maayanlab.cloud/Enrichr/)^39^ enables enrichment analysis of the gene list based on genome-wide experiments. This study utilizes this open-access tool to perform pathway analysis (BioPlanet 2019, KEGG 2021 Human, Elsevier Pathway Collection and Reactome 2022) and ontology analysis of the DEGs categorized into three semantics: Biological Processes (BP), Molecular function (MP) and Cellular Component (CC).

**Figure 1:**
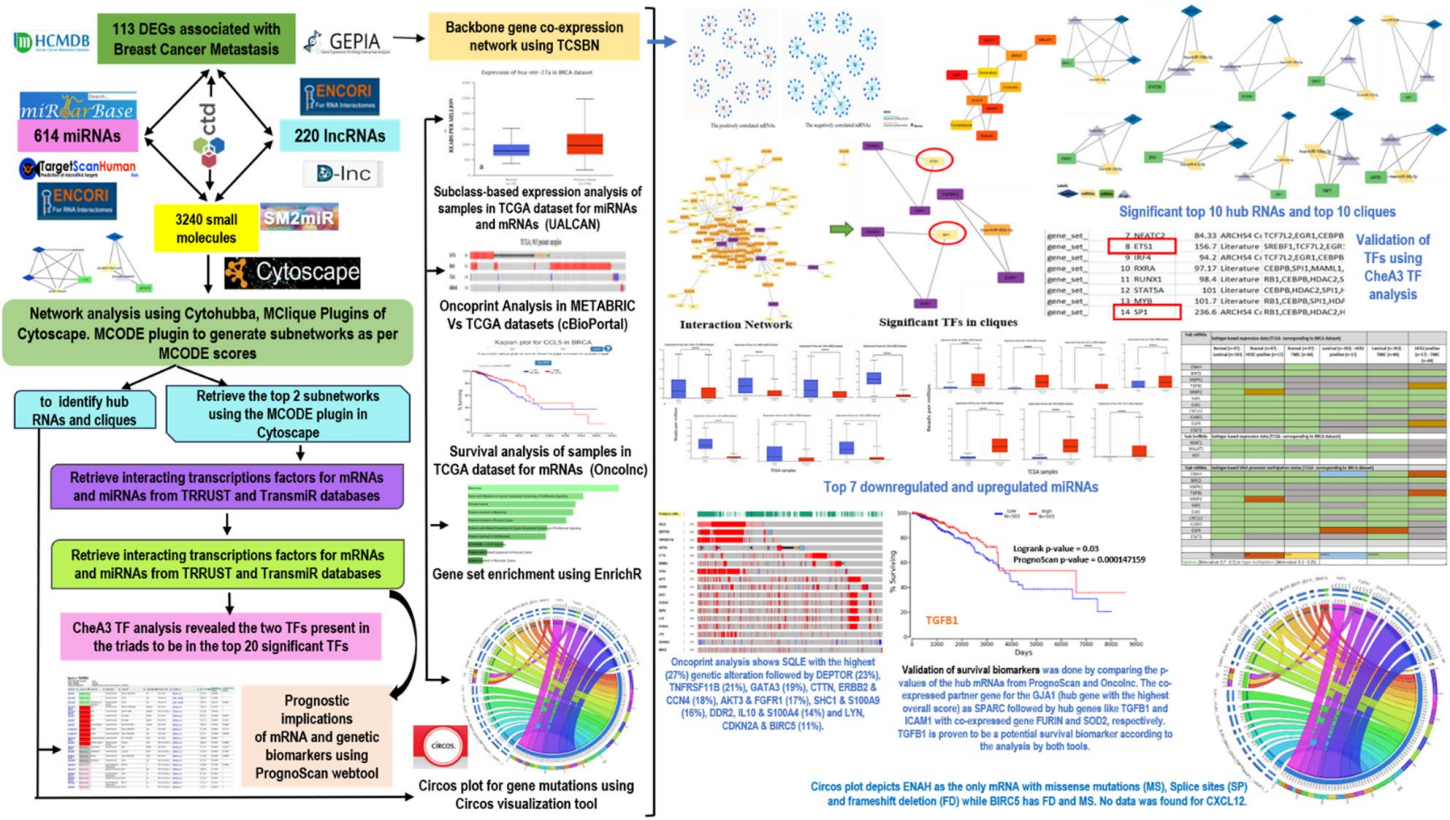
The integrated methodology of the study exploring the role of coding and non-coding RNAs, small molecules and transcription factors involved in breast cancer metastasis.

Figure 1 schematically represents the framework of the overall study.

### Ethical approval

This article does not contain any studies with human participants or animals performed by any of the authors.

## Results

### Obtaining target RNAs and interacting drugs

Initially, 450 BC metastatic mRNAs are retrieved from HCMDB database followed by retrieval of 3159 BC associated differentially expressed mRNAs from GEPIA2. Comparing both the gene lists, 113 differentially expressed mRNAs involved in BC carcinogenesis. These 113 DEGs are found to be interacting with 614 common miRNAs retrieved from TargetScan, ENCORI and miRTarBase databases and 220 common lncRNAs of TarBase, lncBase (mRNA–lncRNA interaction data) and ENCORI (miRNA–lncRNA interaction data) are considered for the study. 1049 drugs interacting with mRNA, miRNA and lncRNA were discovered from CTDbase, Sm2miR and D-lnc database, respectively.

### BC-specific backbone PPI network

**Figure 2:**
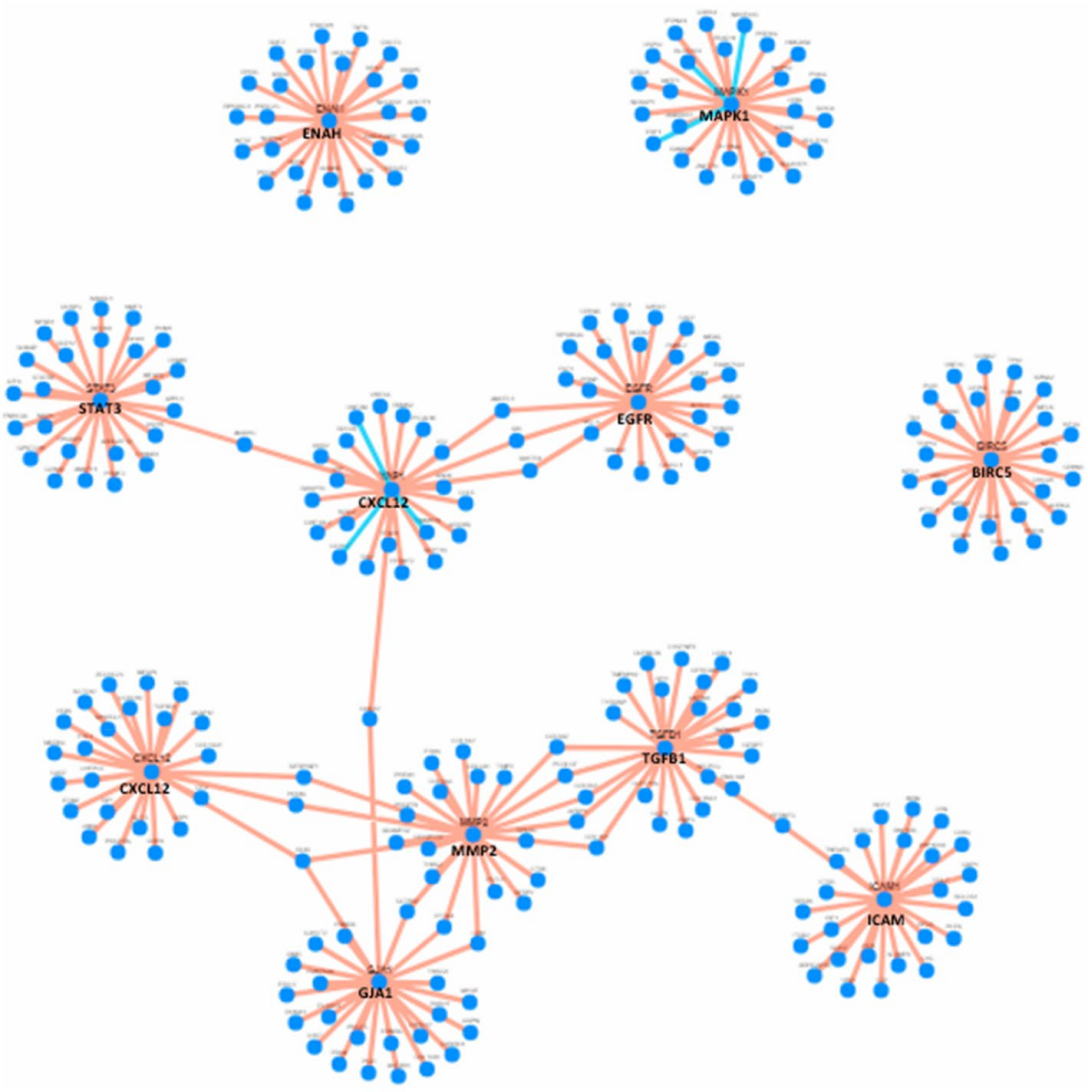
Overall correlation network for the 11 hub mRNAs. The orange lines represent positive correlation and blue lines represent negative correlation of genes. The central nodes are the 11 hub DEGs and the surrounding nodes are correlated genes forming clusters.

The backbone gene co-expression network for the top 20 hub DEGs provides the positively and negatively correlated genes involved in the network (Fig S3) using TCSBN. The correlated gene clusters are listed along with their *p* values in Tables S1 and S2. The positively correlated edges are represented as orange lines while the negative ones as blue lines. The nodes are in blue for both the correlation networks representing genes. The net correlation network is represented in Fig. 2.

### Interaction network analysis using cytoscape v3.9

**Figure 3:**
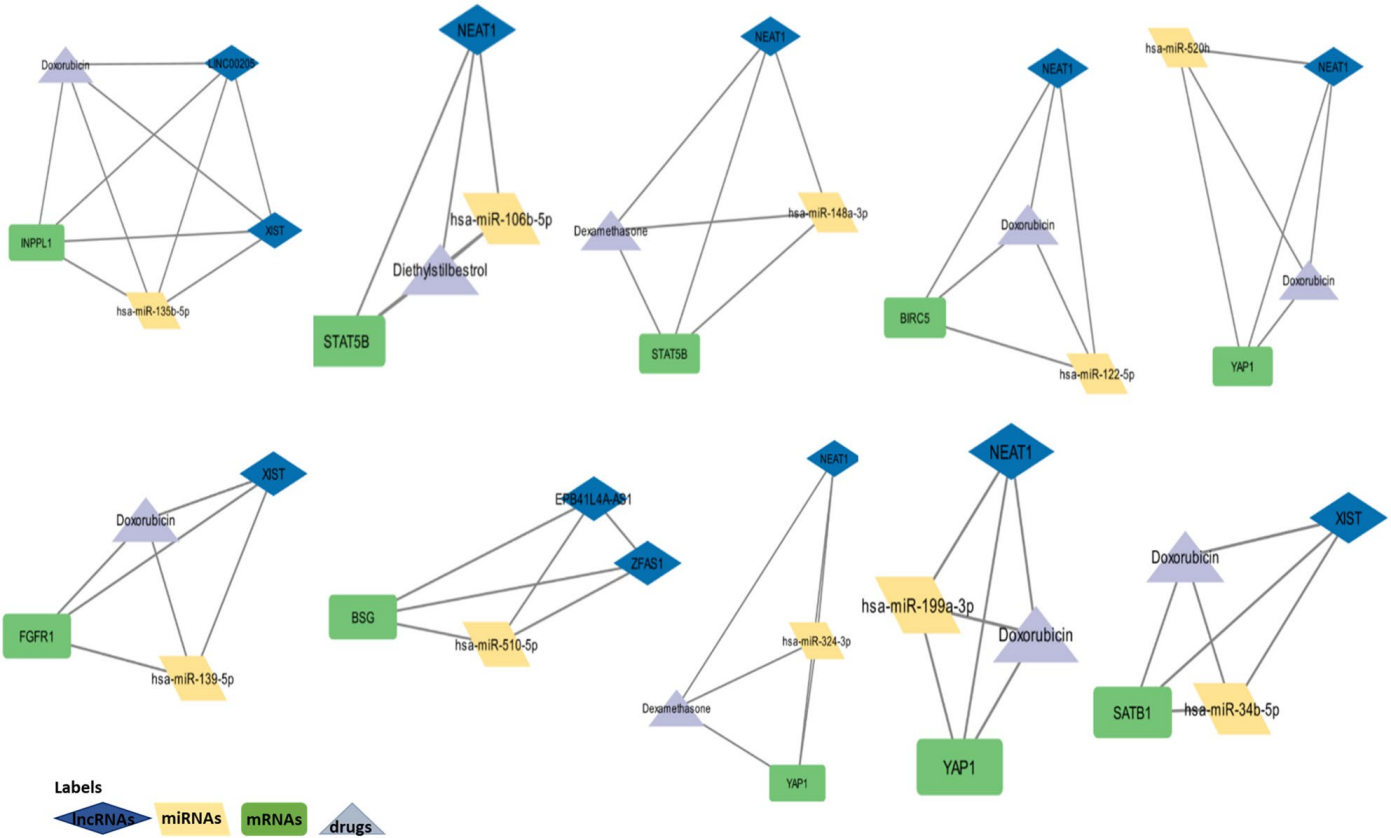
Top 10 cliques from the complex mRNA–miRNA–lncRNA–drug interaction network. DEGs involved in the cliques include INPPL1, STAT5B, BIRC5, YAP1, SATB1, BSG, FGFR1. Significant lncRNAs include NEAT1 (6) and XIST (3). Drugs in maximum number of cliques include doxorubicin (6) and dexamethasone (2). There are no shared miRNAs among the top 10 cliques.

In cytoscape, the interaction data tables are loaded individually summing up to six different interaction tables: mRNA–miRNA, miRNA–lncRNA, mRNA–lncRNA, mRNA–small molecules, miRNA–small molecules and lncRNA–small molecules interaction network. The complex interaction network is generated by merging these individual interaction networks (Fig S1). In the interaction network the mRNAs, miRNAs, lncRNAs and drugs are represented by green rectangles, yellow parallelogram, blue rhombus and grey triangles, respectively. Cytohubba and MClique plugins applied on the interaction network reveals the top 10 hub RNAs (STAT3, MAPK1, BIRC5, NEAT1, XIST, MALAT1, Doxorubicin, Curcumin, Formaldehyde, Glucose) and a total of 656 cliques: one pentagon clique with 5 nodes, 133 cliques with four nodes and remaining 522 cliques comprised of three nodes. Top 10 cliques are represented in Fig. 3. Among the top 10 cliques generated, significant mRNAs include INPPL1, STAT5B, BIRC5, YAP1, SATB1, BSG, FGFR1, significant lncRNAs include NEAT1 and XIST and significant drugs present in the top 10 cliques include doxorubicin (6) and dexamethasone (2). Ten different miRNAs are involved in cliques. No redundant/common miRNAs among the top 10 cliques.

### Retrieval of sub networks from the interaction complex network

**Figure 4:**
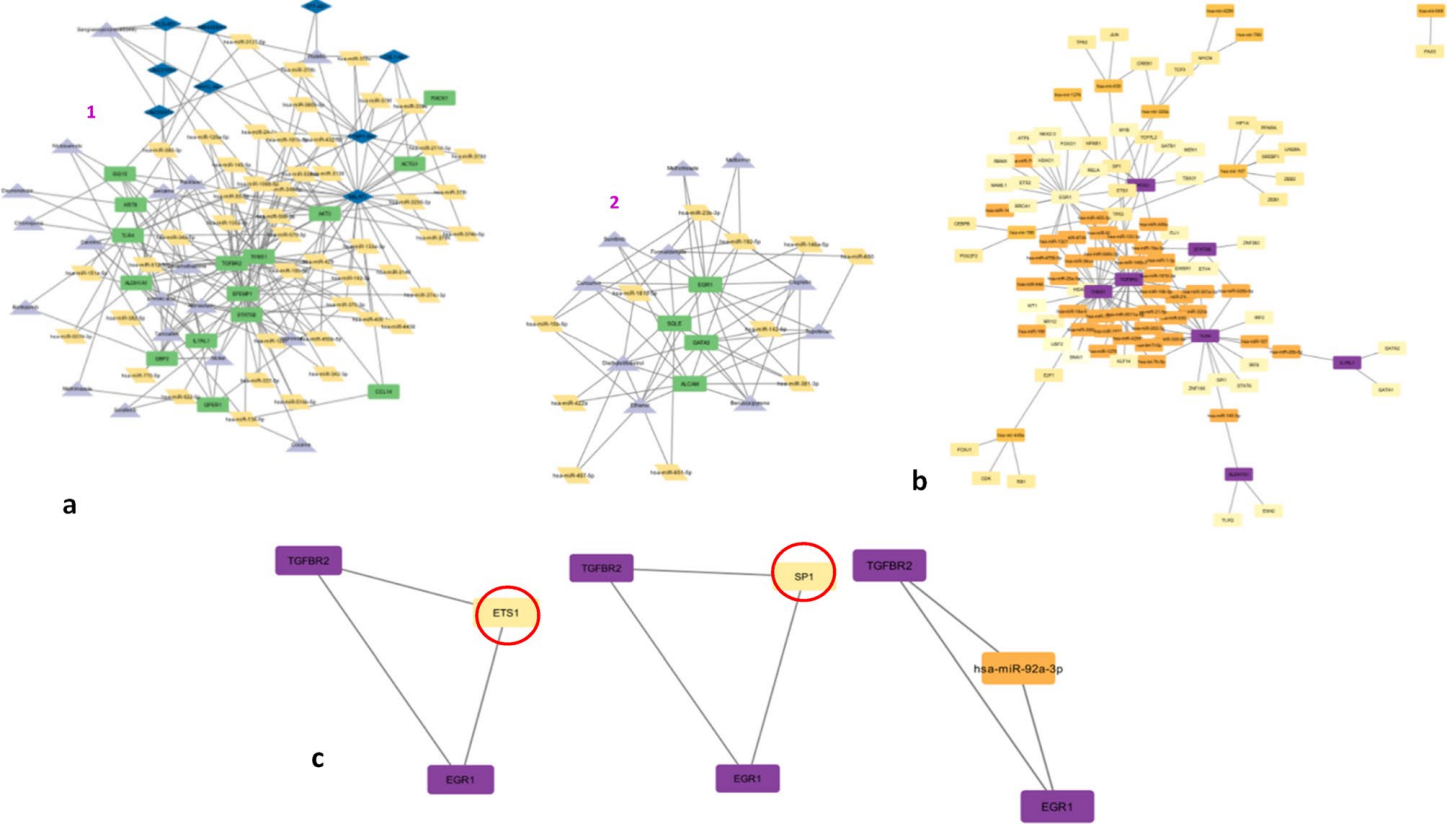
(**a**) Based on the MCODE plugin score of cytoscape, the top 2 sub-networks are retrieved: Cluster 1 with a score of 5.438 and Cluster 2 with a score of 5.417. (**b**) The merged network for mRNAs-miRNA-TF (**c**) Cliques generated involving mRNAs-miRNAs-TFs reveal two significant TFs: SP1 and ETS1.

MCODE is implemented on the interaction network based on the haircut algorithm taking into consideration the parameters like a k core of 2, a node cut-off value of 0.2, and a maximum depth of 100. To create sub-networks, the top two clusters according to the clustering score were utilised (Fig. 4a) taking into consideration the top 20 hub RNAs (Fig S2) using cytoscape. The first module had cluster score of 5.438 encompassing 90 nodes and 242 edges followed by the module with a cluster score of 5.417 that was made up of 25 nodes with 65 edges.

Gene ontology (GO) analysis of the top 2 modules revealed their involvement in salivary gland morphogenesis, lymph node development, pathway restricted SMAD protein phosphorylation, JAK-STAT cascade involved in growth hormone signalling pathway (biological processes), ion transmembrane transported activity and Ran GTPase binding (molecular function) and connexon complex, contractile fiber, pseudopodium, dendrite cytoplasm, axon and gap junction (cellular components) were the topmost ontologies enriched.

### Construction of TF–miRNA–mRNA interaction network and TF analysis

The mRNAs involved in these two sub-networks (Fig. 4a) are further exploited to study a mRNA–miRNA–transcription factor (TF) interaction network. For the 8 common mRNAs involved, 39 interacting miRNAs and 56 interacting TFs were retrieved. The merged network for mRNA–miRNA–TF is generated (Fig. 4b). The MClique plugin of cytoscape is utilised to reveal that two of the 56 TFs (ETS1 and SP1) are present in two of the three generated cliques (Fig. 4c). Two out of the three cliques have TFs ETS1 and SP1. Further, the CheA3 TF analysis reveals that these two TFs from the cliques are present among the top 20 integrated mean rank TF analysis (Table S3). The third clique is comprised of two mRNAs and one miRNA.

### Expression, survival and oncoprint analyses of the DEGs

#### Expression analysis of mRNAs and lncRNAs

*Subclass-based expression analysis.* The expression analysis of mRNAs and miRNAs was performed using the UALCAN tool (Table 1). The BC subtype-based expression analysis reveals GJA1 is the most expressed mRNAs and TGFB1 and EGFR has similar expression levels (Fig S4) in BC subtypes while the hub lncRNAs (NEAT1 and MALAT1) (Fig S5) are almost similarly expressed in all BC subtypes. XIST less expressed in luminal than the other lncRNAs.

**Table 1:**
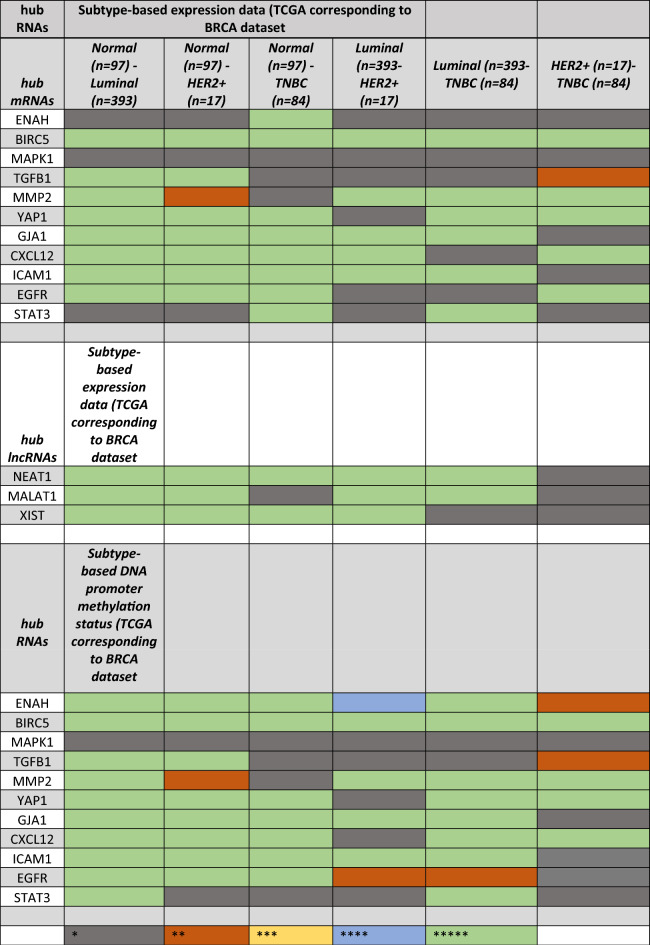
Subclass-based expression analysis of hub RNAs and DNA promoter methylation status of mRNAs.

*Promoter-methylation based expression*. The promoter methylation status analyses of the mRNAs revealed that (Table 1) MMP2, CXCL12, MAPK1, ICAM1 and BIRC5 are significantly methylated in Luminal A subtype of BC. GJA1, ENAH, EGFR, TGFB1and YAP1 are highly methylated in HER2 + BC subtype. STAT3 is the only gene found to be most methylated in triple-negative breast cancer (TNBC).

#### Expression analysis of miRNAs

**Figure 5:**
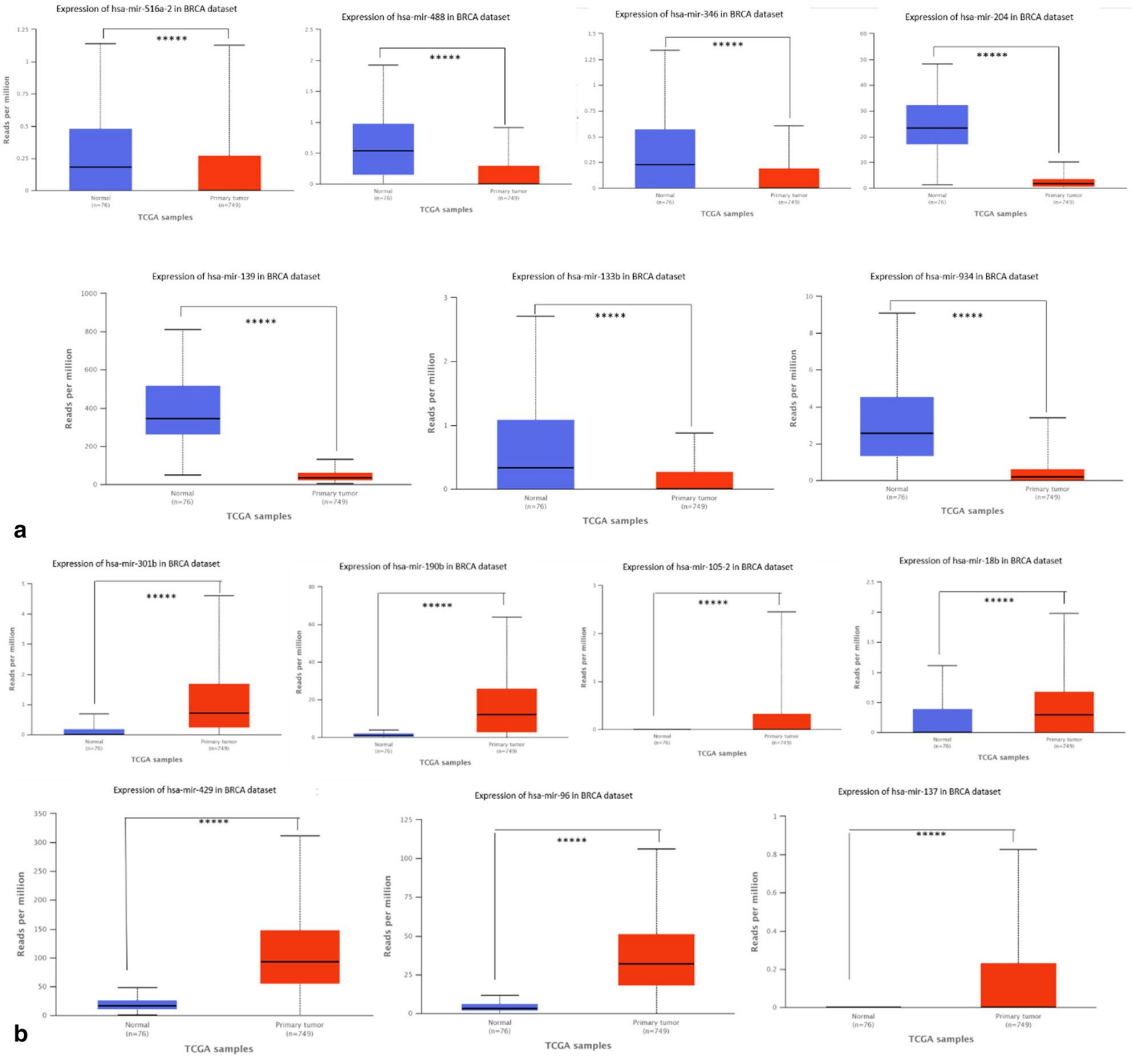
(**a**) Top 7 downregulated miRNAs include miR-516-2, miR-488, miR-346, miR-204, miR-139, miR-133b and miR-934. (**b**) Top 7 upregulated miRNAs include miR-301b, miR-190b, miR-105-2, miR-18b, miR429, miR-96 and miR-137.

Expression levels of miRNAs (Fig. 5a,b) reveals that miR-105-2 and miR137 are the most significantly expressed miRNAs, while miR-204 and miR133b expression levels are reduced in BC (Fig S6).

**Figure 6:**
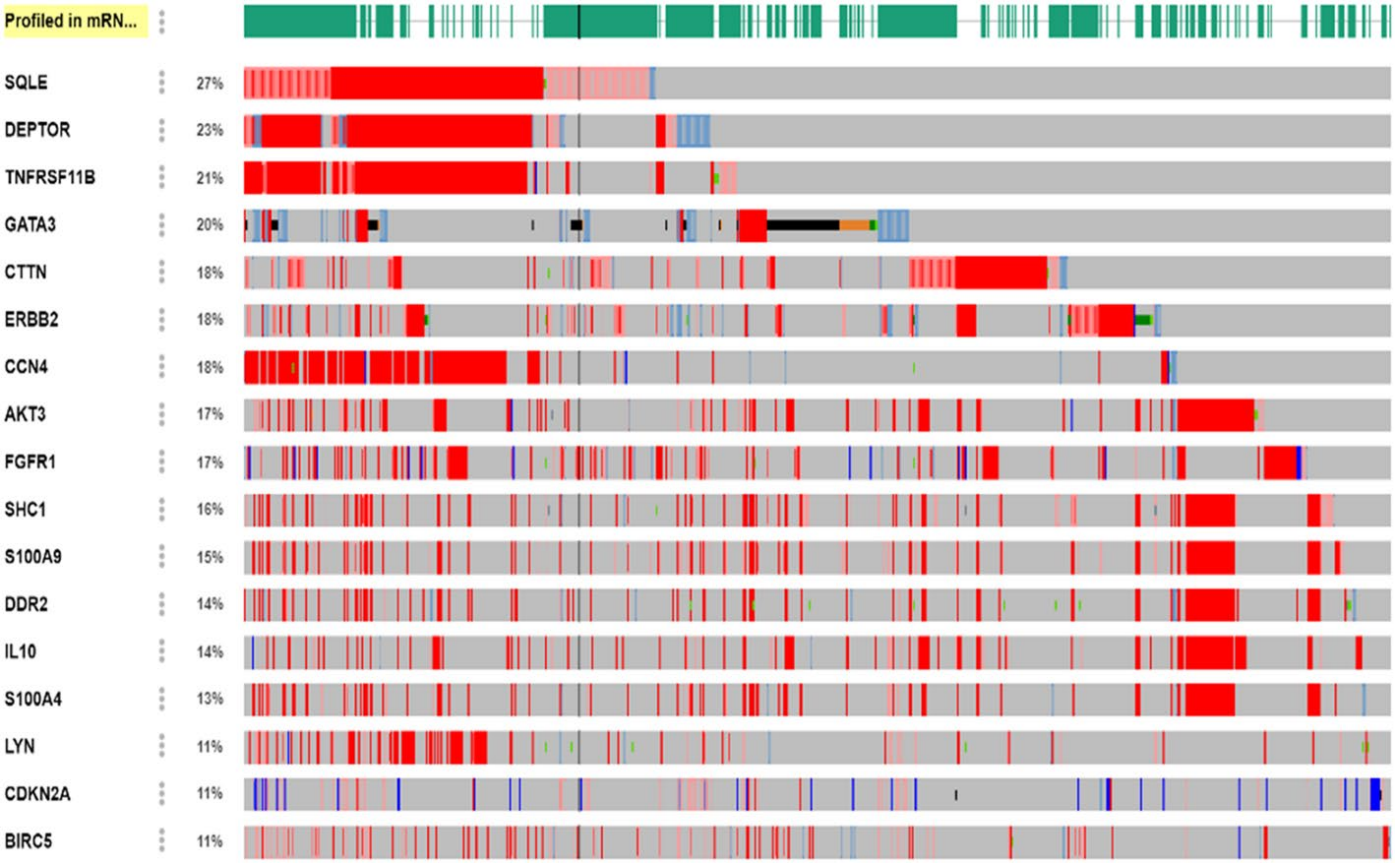
The oncoprint analysis of the significantly altered DEGs. > 10% genetic regulation of SQLE, DEPTOR, TNFRSF11B, GATA3, CTTN, ERBB2 & CCN4, AKT3 & FGFR1, SHC1 & S100A9, DDR2, IL10 & S100A4 and LYN, CDKN2A & BIRC5 from TCGA dataset.

Oncoprint analysis results from cBioPortal reveals that SQLE has the highest (27%) genetic alteration followed by DEPTOR (23%), TNFRSF11B (21%), GATA3 (19%), CTTN, ERBB2 & CCN4 (18%), AKT3 & FGFR1 (17%), SHC1 & S100A9 (16%), DDR2, IL10 & S100A4 (14%) and LYN, CDKN2A & BIRC5 (11%). The oncoprint analysis of all the significant DEGs are depicted in Fig. 6.

### Survival Analysis of DEGs

Survival Analysis using Oncolnc in Table 2 lists significant survival biomarkers with log rank *p* values of less than 0.05. Further, *p* values from the PrognoScan tool analysis and Fp class scores validates the survival status of the top hub mRNAs.

**Table 2:** Kaplan–Meier plot analysis of DEGs significant in survival of BC patient.

Survival biomarkers	Logrank *p* value
ADGRF5	0.0379
ALDH1A1	0.0284
FOXM1	0.0125
GBP2	0.0221
KRT5	0.0442
STAT5A	0.0163
TGFB1	0.03

Validation of survival biomarkers was done by comparing the *p* values of the hub mRNAs from PrognoScan and Oncolnc (Table 3). This was done to finalize the most probable survival biomarkers that can be targeted as a therapeutic alternative to manage BC. To each hub gene, a gene expression score and a network’s topological score were generated. The co-expressed partner gene with the common survival marker TGFB1 (hub gene) was identified as FURIN.

**Table 3:** Validation of top 20 hub genes: by utilizing the *p* value from PrognoScan and Oncolnc and Fp Class scores.

hub mRNA	PrognoScan	Oncolnc	Fp class			
			Co-expressed gene symbol	Total score	Gene co-expression score	Network topology score
BIRC5	0.000296436	0.07	NUSAP1	0.9429	0.9423	0
STAT3	0.0027682	0.1	SOD2 (Superoxide dismutase [Mn], mitochondrial)	0.5549	0.527	0
MAPK1	9.00E-06	0.5	KCTD20	0.4916	0.4457	0
TGFB1	0.000147159	0.03	FURIN	0.4944	0.1083	0
MMP2	0.00829304	1	COL1A2	0.8826	0.8801	0
EGFR	3.39E-05	0.3	FBLN1	0.8117	0.6334	0
ENAH	0.00351316	0.5	PTK2	0.2609	0.1297	0
GJA1	0.000256725	0.04	SPARC	0.7426	0.731	0
CXCL12	3.02E-05	0.3	DCN	0.8826	0.8436	0
YAP1	2.71E-05	0.3	ID1	0.803	0.0741	0
ICAM1	0.00176679	0.03	SOD2 (Superoxide dismutase [Mn], mitochondrial)	0.5027	0.4925	0

### Mutation-related data for DEGs

**Figure 7:**
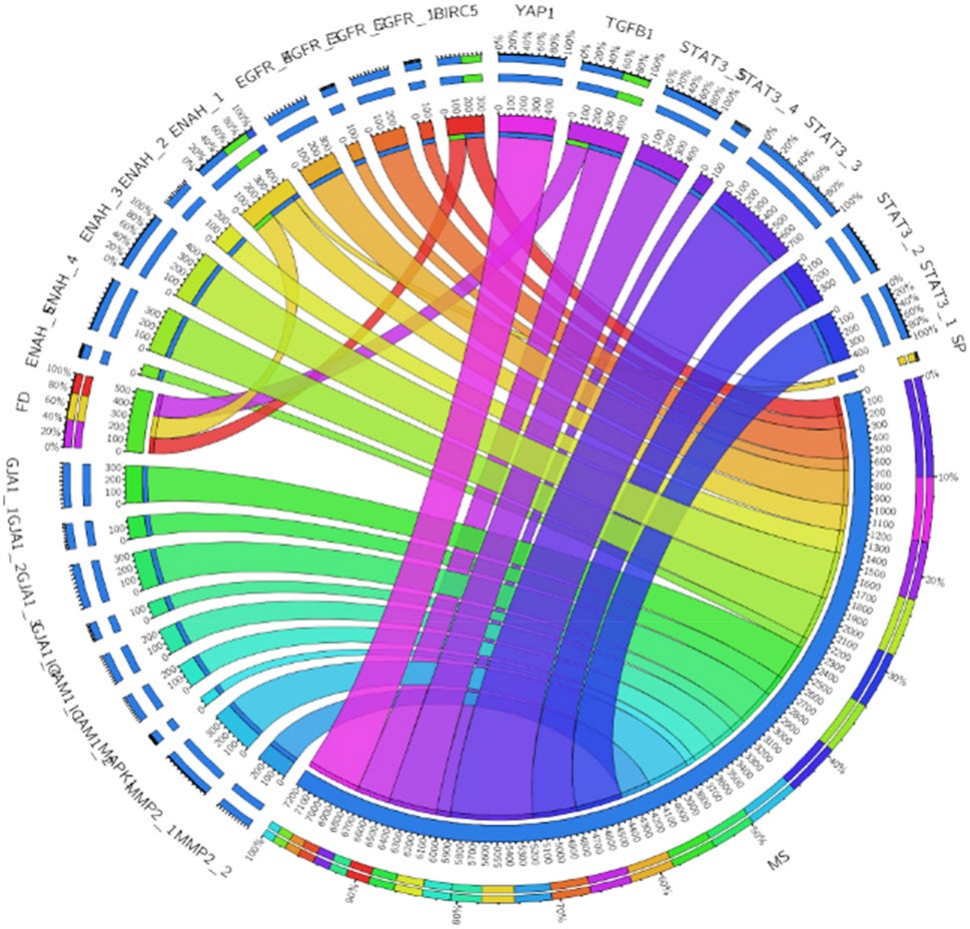
Circos plot representing mutations of the 11 DEGs. FI-frameshift insertions, FD-frameshift deletions, SP-splicing.

From cBioPortal, the types of mutation for each hub mRNAs are obtained. Circos plot (Fig. 7) represents missense, frameshift deletions and splicing in different chromosomal positions of the hub genes.

The circular two-dimensional graphical representations known as “circos plots” offer a thorough method for presenting and understanding multi-dimensional genetic data. The different versions of the same genes are mentioned as gene_1, gene_2 and so on depending on the number of different chromosomal positions at which the mutations occur. ENAH is the only mRNA with missense (MS), Splice sites (SP) and frameshift deletion (FD) while BIRC5 has FD AND missense mutations. No data was found for CXCL12. The Table S4 lists all the mutations with respective chromosomal positions for the 11 hub DEGs.

### Enrichment analysis of DEGs

**Figure 8:**
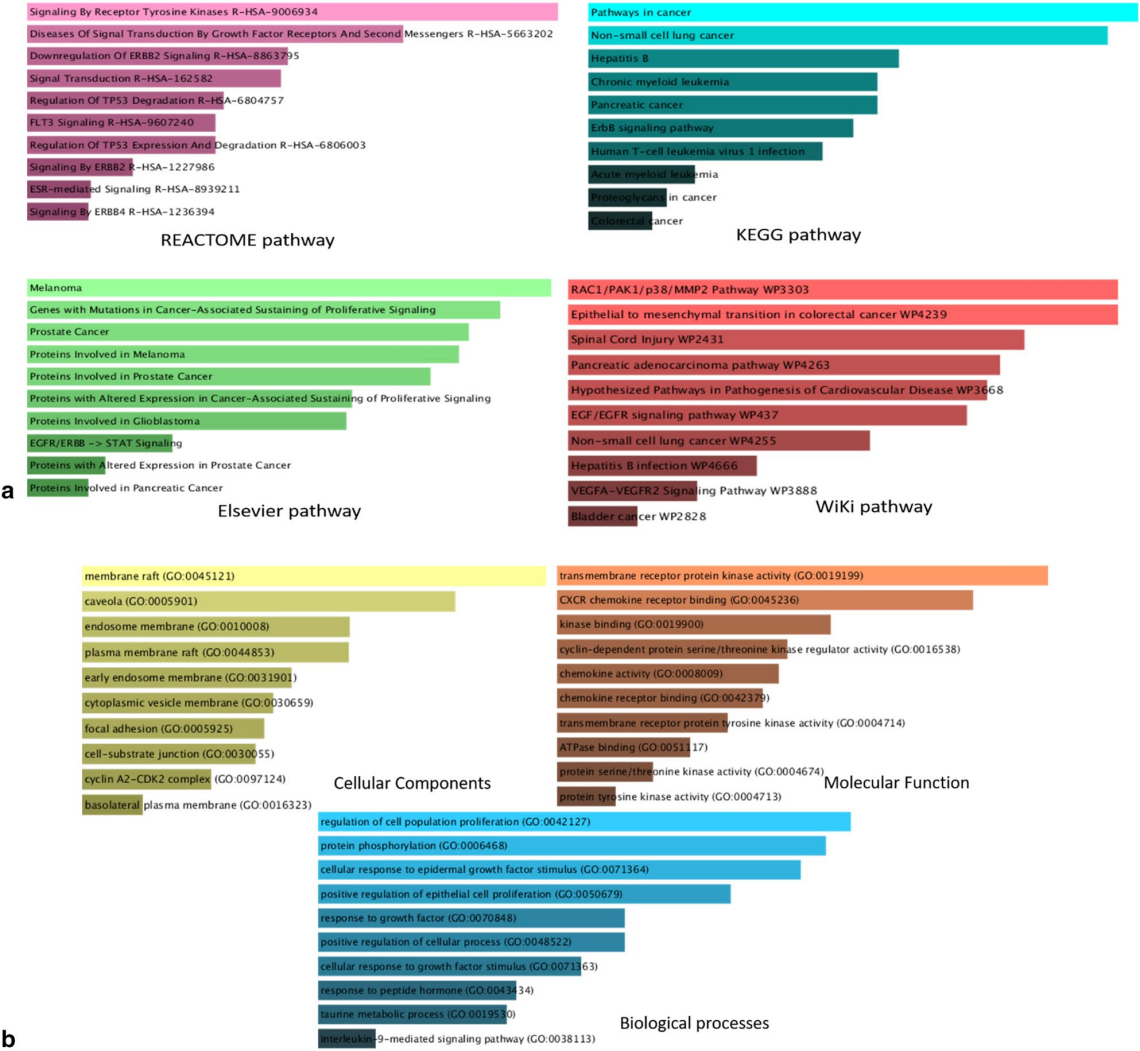
(**a**) Pathways enriched by the 113 DEGs as per REACTOME, KEGG, Elsevier and WiKi datasets. (**b**) Ontology enrichment (cellular components, molecular function and biological processes) of the 113 DEGs.

The web-based EnrichR tool (Fig. 8a,b) is used for pathway enrichment and functional ontology analysis. Based on REACTOME, KEGG, Elsevier and WiKi datasets, the most significant pathways include ‘Role of ERBB2 in Signal Transduction and Oncology’, ‘Bioactive Peptide Induced Signaling Pathway’, ‘IL-2 Receptor Beta Chain in T cell Activation’, ‘TPO Signaling Pathway’ and ‘cell cycle arrest at G1/S Check point’ to name a few.

Transmembrane receptor protein kinase activity, transmembrane receptor protein tyrosine kinase activity, protein tyrosine kinase activity, type I transforming growth factor beta receptor binding and serine-type peptidase activity are the top 5 molecular functions that are regulated by mRNAs like AXL, FLT4, ERBB2, EGFR, TGFBR2, DDR2, FGFR1, TGFB1, ENG, TGFBR2, ST14, HGF, HTRA3, MMP2, MST1 and F3.

TGFB1, ANGPT2, MMP2, MST1, F3, THBS1, SOD3, ICAM1, VCAN, EFEMP1, CXCL12, TIMP3, S100A4, S100A9, TGM2, CAV1, ITGA2, EGFR, ENAH, FERMT1, IL1RL1, GJA1, ALCAM, CTTN, BSG, MAPK1, TGM2, DDR2 and ENG are some of the mRNAs that are present in the collagen-containing extracellular matrix, cell-substrate junction, focal adhesion points, intracellular membrane-bounded organelle and integral component of plasma membrane amongst many. The top significant biological processes include cytokine-mediated signaling pathway, cellular response to cytokine stimulus, regulation of cell population proliferation, positive regulation of intracellular signal transduction and positive regulation of cell population proliferation.

## Discussion

Conventionally, an individual signaling pathway or a dysregulated proteins is targeted for devising therapies. In this work, DEGs involved in BC are considered that mediate the functioning of various ncRNAs involved in the disease development. Hence, we have utilised network-based approaches to identify specific cliques involving the interaction of DE mRNAs, miRNAs, lncRNAs and drugs/small molecules that can be considered for devising newer therapeutic strategies as they give us an overall idea of the disease environment under the influence of interacting RNAs and drugs. The ncRNAs are often not extensively discussed or considered with respect to disease causing phenomena and in some of the studies they are considered as separate identities, when the underlying mechanism of disease involves the interaction of mRNAs and ncRNAs with each other. For instance, when no drug is provided into a system, dysregulated levels of ncRNAs can modulate the levels of coding RNAs or expression of protein levels leading to a diseased state. Through this network-based approach we are trying to understand the crosstalk between mRNAs and ncRNAs that can therapeutically be addressed in future considering the entire disease environment. Differential expression of genes helps in the developing in-depth biological insight into the underlying mechanism of a disease condition owing to its interaction with various ncRNAs and drugs that also play a major role in modulating the overall scenario underpinning any diseased state.

According to the in-silico study conducted, 11 out of 113 differentially expressed metastatic genes undergo different types of mutations, primarily missense, frameshift and splicing that lead to the diseased state. Regulation of expression of different DEGs in turn influence the expression levels of miRNAs, lncRNAs and different molecules (small molecules/drugs). Among the mRNAs from the top 10 cliques generated, BIRC5 was one of the most significant with more than 10% of probable genetic alteration. BIRC5 expression is associated with resistance to chemotherapy, radiation and neo-adjuvant therapy, specifically in II/III stage of BC^40^. Also, with age of the patients the expression of BIRC5 increases and hence targeting it aids in better survival in BC patients. Significant amount of DNA methylation is observed in BC tissues. Also, from the DNA promoter methylation status of BIRC5 reveals its negative association with the methylation status of the gene. Survivin coded by BIRC5 is responsible for cell division bypassing the cell death in normal as well as cancer cells leading to decreases survival of cancer patients.

However, the validated survival markers include TGFB1, GJA1 and ICAM1. The role of TGFB1 is multifaceted in BC depending on the stage of cancer^41^. This cytokine exhibits tumor-suppressing properties in the initial stages of BC by hindering epithelial cell cycle progression and enhancing apoptosis. TGFB1 is associated with tumor development, higher cell motility, cancer invasiveness, and metastasis in late stages, however. Additionally, it promotes the EMT and modifies the cancer microenvironment. However, there are three known therapeutic strategies involving TGFB1. On order to disrupt ligand-receptor interactions, soluble receptors and anti-receptor monoclonal antibodies are used along with TGFB1 receptor kinase inhibitors and peptide aptamers that suppress intracellular signalling cascades by using antisense compounds to impede TGFB1 production on the ligand level^42–45^. GJA1 is subtype-dependent mRNA that codes for the protein Connexin-43 (Cx43). Cx43 is overexpressed in ERα- or PR-positive breast tumors compared to ERα- or PR-negative tumors^46^. The expression of ICAM1 is directly proportional to the metastatic potential of the tumor cells. Reduced invasion of human epithelial BC cells in vitro is done by targeting endogenous human ICAM1^46^.

The significant DEGs are not only identified by exploiting multiple correction algorithms but are also backed up by existing literature review regarding their relevant role in BC. Hence, the results are consistent and comparable (Table 4) as per literature provided regarding the role of these DEGs in BC.

**Table 4:** Comparison of our integrated analysis with similar studies reported in the literature.

Cancer type	Gene-expression	mRNA–miRNA–lncRNA–drugs	Hub identification	Functional module /subnetwork extraction	Pathway enrichment analysis	Oncoprint and survival analysis	Mutation circos plots	References
Colon cancer	Yes	No	Yes	Yes	Yes	Yes	No	^47^
Oral cancer	Yes	No	Yes	Yes	Yes	Yes	No	^48^
Cervical cancer	Yes	No	Yes	Yes	Yes	Yes	No	^49^
Triple-negative breast cancer	Yes	No	Yes	Yes	Yes	Yes	No	^50^
Breast cancer	No	No	Yes	No	Yes	No		^51^
benzo[a]pyrene-treated breast cancer cells	No	No	No	No	Yes	No	No	^52^
Gastric cancer	Yes	No	Yes	Yes	Yes	No	No	^53^
Hepatocellular carcinoma	Yes	No	Yes	Yes	Yes	No	No	^54^
Prostate cancer	No	No	No	Yes	Yes	No	No	^55^
Breast cancer	Yes	Yes	Yes	Yes	Yes	Yes	Yes	This study

ZFAS1 is generally known to be a tumor suppressor lncRNA. Overexpressed levels of ZFAS1 are associated with decreased tumor cell proliferation leading to apoptosis of BC cells. In addition to this, significant expression levels of ZFAS1 leads to decreased metastasis by regulating EMT^56^. ZFAS1 binds with the CDK1/cyclin B1 complex leading to destabilized state of p53, which promotes cell cycle advancement and inhibits apoptosis^57^. The expression levels of ZFAS1 is in human BCs is much less compared to its levels in normal native tissues^58^. Upregulated levels of ZFAS1 in BC targets miR-589 by the PTEN/PI3K/AKT signal pathway modulation resulting in possible inhibition of proliferation, tissue invasion and metastasis of BC cells. The role of linc00205 that is the most shared lncRNA among the 636 cliques generated. The role of this lncRNA is not quite explored in BC. It is known to promote tumorigenesis and metastasis by competitively suppressing miRNA-26a in gastric cancer^59^. Additionally, it speeds up the growth of hepatoblastoma via controlling the microRNA-154-3p/Rho-associated kinase 1 axis through mitogen-activated protein kinase signalling. WDFY3-AS2 is found to be decreased in TNBC and hence serves as a potential prognostic factor in TNBC development^60^ while its overexpression is associated with inhibition of tumor cell growth, cell migration and invasion^61^.

Due to its inherent and extrinsic propensities to suppress carcinogenesis, the TF ETS1 may be an effective therapeutic target for BRCA^62^. SP1 interacts with the insulin-like growth factor I receptor to regulate BC proliferation. Additionally, SP1 promotes angiogenesis by binding to the VEGF promoter, creating a favorable environment for the development of tumors.

Among the top upregulated and downregulated miRNAs there are miR-105 and miR-137 and miR204 and miR934, respectively. miR-105 has an intricate function in the onset and propagation of cancer. Given the specific tumor setting and the pairing of bases in genes, miR-105 either functions as a tumor suppressor by preventing metastasis or as an oncogene by encouraging tumor initiation and tissue invasion^63^. Evidence suggests that miR-137 plays a function in tumor suppression via altering Del-1 expression in TNBC^64^. In breast tissues, miR-204-5p was dramatically downregulated, and patients with BC who expressed more of it had better survival rates^65^. miR-934 mediated regulation of PTEN and EMT results to BC metastasis^66^.

When it comes to the small molecules or drugs found in the top 10 cliques, it is seen that they are approved drugs that are clinically available to treat BC. The drugs from the top 10 significant cliques like doxorubicin, dexamethasone are the conventional drugs used for BC treatment. As mentioned earlier, these drugs come with various side effects alongside drug resistance by BCCs. Owing to interaction with approved drugs, not only mRNAs, miRNAs and lncRNAs act as potential candidates but also the two TFs that are within the top 14 in ChEA3 analysis when targeted have the ability to treat BC. Targeting such ncRNAs and TFs can help in dosage modulations of such conventional drugs reducing adverse effects and hence add a therapeutic value into the BC regimen.

### Future prospects of the interaction study

The hERG channel activity is vital for normal cardiac functioning. Any drug-mediated hindrance in the channel activity leads to serious cardiotoxicity resulting to prolonged QT interval. Hence it is of utmost need to evaluate the role of drug molecules in modulating the channel activity. In a study^67^ dealing with cardiotoxicity imparted by the hERG channel blockers, a robust deep learning (DL) model called DMFGAM is utilised. It is a fivefold experimentally cross validated model based on the molecular fingerprints and graph attention mechanism. This model serves as a significant tool to assess hERG channel blockers in initial phases of drug discovery and development.

Similar to network-based approach, newer technologies are needed to be developed to understand the relationships among various bioentities involved in a disease. In a study by Sun et al.^68^, a novel DL algorithm named as ‘graph convolutional network with graph attention network’ (GCNAT) to predict the potential associations of disease-related metabolites. The graph convolutional neural network is used to encode and learn characteristics of metabolites and diseases. The encapsulations of several convolutional layers are then combined using a graph attention layer, and the associated attention coefficients are determined to give the embeddings of each layer various weights. The final synthetic embeddings are decoded and scored in order to achieve the prediction result. Finally, GCNAT surpasses the outcomes of the current five state-of-the-art predicting algorithms in fivefold cross-validation, achieving a dependable area under the receiver operating characteristic curve of 0.95 and a precision-recall curve of 0.405.

GCNCRF^69^ is a technique for predicting human lncRNA–miRNA interactions that is based on the graph convolutional neural (GCN) network and conditional random field (CRF). Using the LncRNASNP2 database’s known lncRNA and miRNA interactions, the lncRNA/miRNA integration similarity network, and the lncRNA/miRNA feature matrix, we first build an eclectic network. Second, a GCN network is used to obtain the first embedding of nodes. The generated initial embeddings can be updated by a CRF set in the GCN hidden layer to ensure that related nodes have similar embeddings. The decoding layer is then used to decode and score the final embedding. GCNCRF achieved a fivefold cross-validation experiment area under the receiver operating characteristic curve value of 0.947 on the primary dataset, outperforming the other six cutting-edge approaches in terms of prediction accuracy.

In a study by Xu et al., it was investigated how components are built in messenger RNAs mRNAs-driven protein droplets with respect to various physical features by developing a Cahn–Hilliard phase-field model coupled with Ginzburg–Landau free-energy scheme. It was observed that the growth rate of droplet size and the assembly of higher-order complexes in a droplet are severally determined by the diffusion rate of the droplet and the binding rate of mRNA with protein. This was done by analyzing the intra droplet hetero patterning of two specific droplets (mRNA- and mRNA-driven droplets). A phase-field model based on the Cahn–Hilliard diffuse interface model to investigate how mRNAs regulate protein phase separation. Whi3 protein is combined with a particular kind of mRNA, which can bind to Whi3 through RRM to create complexes^70^.

In a work by Xiang Li et al.^71^, they assessed exact quantities of up to hundreds of proteins involved in the dynamic assembly and disassembly of TNF signaling complexes using the SWATH-MS approach. When we combined experimental validation with SWATH-MS-based network modelling, we discovered that the cell only experiences TRADD-dependent apoptosis when RIP1 levels are below 1000 molecules/cell (mpc). There is a biphasic relationship between the amount of RIP1 and the occurrence of necroptosis or total cell death. In order to allow RIP1 to play a variety of roles in controlling cell fate decisions, our study offers a resource for encoding the complexity of TNF signalling as well as a quantitative description of how different dynamic interactions between proteins serve as basis sets in signalling complexes.

Recent studies show that inflammasome-activated caspase-3 can trigger secondary necrosis/pyroptosis, which releases fewer inflammatory cytokines and reduces the occurrence of severe immune diseases. GSDME can prevent tumor growth by enhancing cell antitumor function. However, GSDME-induced secondary pyroptosis appears to be minimal in GSDMD- or caspase-1-deficient RAW-asc cells. Further analysis using cells with high GSDME expression, such as bone marrow-derived macrophages (BMDM), is needed to fully understand the role of secondary pyroptosis in these cells. Pyroptosis decreases cell death contribution, while apoptosis becomes important with reduced caspase-1 or GSDMD levels, with low caspase-1 thresholds^72^.

A study^73^ presents a novel matrix factorization model called LMFNRLMI, which predicts lncRNA–miRNA interactions using known positive samples. The model outperforms other models in leave-one-out and fivefold cross validation, improving performance and confirming its superiority. The model aims to be a useful tool for identifying potential lncRNA–miRNA association identification in the future.

Deep Parametric Inference (DPI) is a powerful single-cell multimodal analysis framework that transforms multimodal data into a multimodal parameter space. It can characterize cell heterogeneity more comprehensively than individual modalities and has superior performance compared to state-of-the-art methods. DPI successfully analyzes COVID-19 disease progression in peripheral blood mononuclear cells and proposes a cell state vector field for bone marrow cell states^74^.

A study by Li Zhang et al.^75^ developed a network distance analysis model for predicting lncRNA–miRNA associations (NDALMA) using Gaussian interaction profile (GIP) kernel similarity. The model achieved satisfactory results in fivefold cross validation. NDALMA showed superior prediction performance compared to other network algorithms. Case studies confirmed its reliability in predicting lncRNA–miRNA associations.

Gene function and protein association (GFPA)^76^ is a new analysis framework that mines reliable associations between gene function and cell surface protein from single-cell multimodal data. It reveals cellular heterogeneity at the protein level, demonstrating its reliability across multiple cell subtypes and PBMC samples.

## Conclusion

Clique identification from a complex network of differentially expressed metastatic targets, specifically ncRNAs involved in BC can be explored as the potential biomarkers for the BC. As per ChEA3 analysis, EST1 and SP1 are among the top 14 TFs. As per the BRCA dataset. This study gives an integrated environment scenario of the interaction of the RNAs and their roles in BC metastasis. The BC subtype-based expression analysis reveals TGFB1 and GJA1 are two most expressed mRNAs and can be explored further with respect to its modulatory effects on metastasis and BC stemness. The three validated significant survival markers are TGFB1, GJA1 and ICAM1 having frameshift and missense mutations primarily and hence that can be targeted to aim better overall survival in patients. BIRC5 is one of the key regulators as per the network analysis with significant genetic alteration. This gene is hypermethylated in luminal A and HER2 + ve subtypes. miR-105 and miR-204 are oncomiRs while miR-137 and miR-934 are tumor suppressor miRs. Among the lncRNAs, ZFAS1 and WDFY3-AS1 are tumor suppressor lncRNAs while lnc00205 is an oncogenic miRNA. As tabulated in Table 4, similar researches have been done involving interaction network of different RNAs, but every study lacks one of the other aspects. In this study, the integration is done involving coding as well as ncRNAs along with small molecules/drugs. In addition to various analyses performed, a plot depicting the mutations in BC metastasis for the hub genes is also given in this work. The network-based approach to identify cliques from the complex network utilizes interaction among competitive endogenous RNAs (ceRNAs) to devise a newer therapeutic strategy to treat BC.

### Supplementary Information

Supplementary Information.

## Data Availability

The authors confirm that the data supporting the findings of this study are available within the article and its supplementary and the publicly available data sources used have been mentioned in the manuscript. HCMDB database (https://hcmdb.i-sanger.com/). Gene Expression Omnibus (GEO) (https://www.ncbi.nlm.nih.gov/geo/). GEPIA database (http://gepia.cancer-pku.cn/). ENCORI (https://starbase.sysu.edu.cn/). miRTarBase (https://mirtarbase.cuhk.edu.cn/~miRTarBase/miRTarBase_2022/php/search.php). TargetScan (http://www.targetscan.org/vert_80/). TarBase v8 (https://dianalab.e-ce.uth.gr/html/diana/web/index.php?r=tarbasev8/index). lncBase v3 (https://diana.e-ce.uth.gr/lncbasev3). Comparative Toxicogenomics Database (https://ctdbase.org/). D-lnc database (http://www.jianglab.cn/D-lnc/). ChA3 (https://maayanlab.cloud/chea3/). Tissue/Cancer-Specific Biological Networks (https://inetmodels.com/). UALCAN (http://ualcan.path.uab.edu/cgi-bin/ualcan-res.pl). Oncolnc (http://www.oncolnc.org/). PrognoScan (http://dna00.bio.kyutech.ac.jp/PrognoScan/). Fp tool (http://dcv.uhnres.utoronto.ca/FPCLASS). cBioPortal server (https://www.cbioportal.org/). Circos tool (http://mkweb.bcgsc.ca/tableviewer/-). EnrichR (https://maayanlab.cloud/Enrichr/).

## Abbreviations

BC: Breast cancer
ncRNA: Noncoding RNA
miRNA: MicroRNA
mRNA: Messenger RNA
lncRNA: Long noncoding RNA
EMT: Epithelial-to-mesenchymal transition
NEAT1: Nuclear enriched abundant transcript 1
CCND1: Cyclin D1
LINC01355: Long intergenic non-protein coding RNA 1355
DEGs: Differentially expressed genes
GEO: Gene expression omnibus
TCGA: The cancer genome atlas
HCMDB: Human cancer metastasis database
GEPIA: Gene expression profiling interactive analysis
CTDbase: Comparative toxicogenomics database
MCC: Maximal clique centrality
TF: Transcription factor
GO: Gene ontology
TCSBN: Database of tissue and cancer-specific biological networks
TNBC: Triple-negative breast cancer
MS: Missense
SP: Splice sites
FD: Frameshift deletion
