# Supplementary material for: Multidimensional computational study to understand non-coding RNA interactions in breast cancer metastasis
Source: Sci Rep. 2023 Sep 22;13:15771. doi: 10.1038/s41598-023-42904-6 (PMC10516999; doi:10.1038/s41598-023-42904-6)
Supplement: Supplementary file 1 — Supplementary Information. [file 41598_2023_42904_MOESM1_ESM.docx]

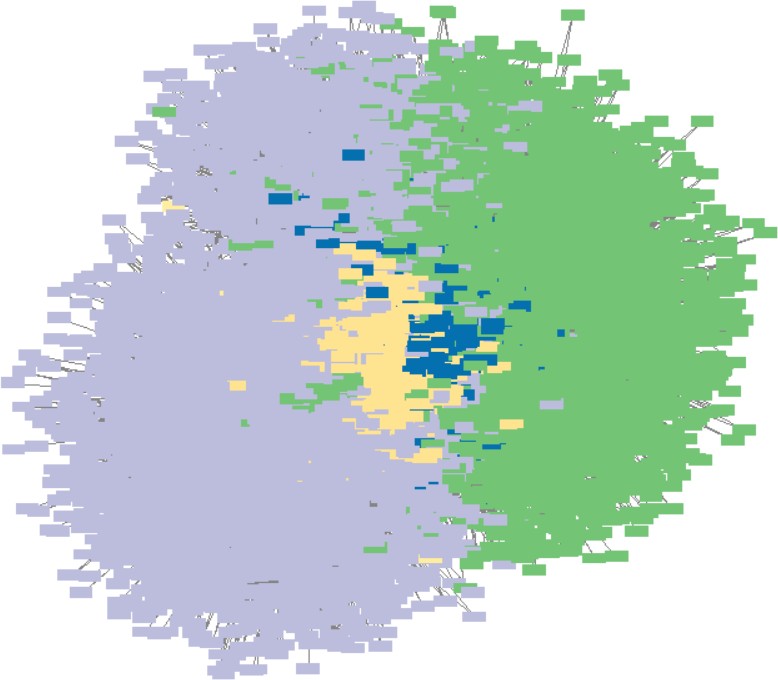
SUPPLEMETARY FIGURES

**Fig S1**. Comprehensive interaction network mRNA-miRNA-lncRNA-drug network


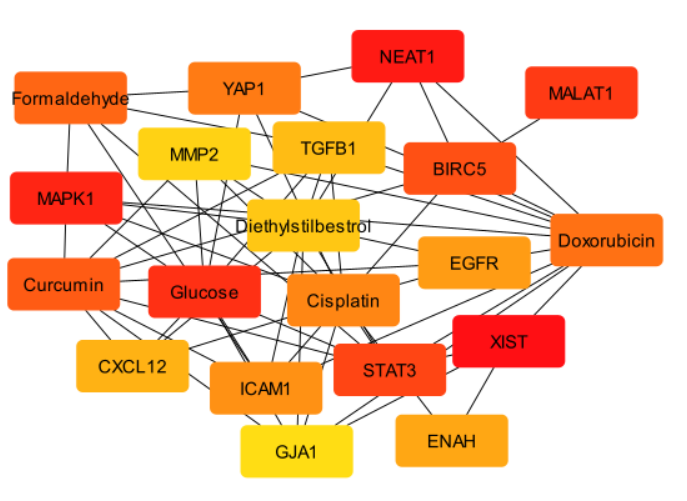


**Fig S2**. Top 20 hub RNAs from cytoscape results

**Table S1.** Positively correlated genes from TCSBN

| **mRNAs** | **correlated mRNAs** | **correlation** | **p-value** | **adj p-value** |
| --- | --- | --- | --- | --- |
| EGFR | SFRP1 | 0.786 | 9.50E-257 | 7.15E-253 |
| EGFR | MET | 0.768 | 1.44E-238 | 7.73E-235 |
| MRAS | EGFR | 0.747 | 4.29E-218 | 1.56E-214 |
| RBMS1 | EGFR | 0.746 | 8.33E-218 | 3.01E-214 |
| EGFR | FOXC1 | 0.744 | 1.18E-215 | 4.09E-212 |

| FAM171A1 | EGFR | 0.743 | 4.06E-215 | 1.39E-211 |
| --- | --- | --- | --- | --- |
| PRNP | EGFR | 0.741 | 7.98E-213 | 2.60E-209 |
| EGFR | CAV2 | 0.736 | 3.51E-209 | 1.06E-205 |
| EGFR | RNF145 | 0.731 | 5.56E-205 | 1.55E-201 |
| EGFR | WWTR1 | 0.724 | 2.91E-199 | 7.16E-196 |
| MAPK1 | CRKL | 0.723 | 2.93E-198 | 7.09E-195 |
| EP300 | MAPK1 | 0.695 | 2.44E-177 | 3.70E-174 |
| PRR14L | MAPK1 | 0.675 | 4.27E-163 | 4.53E-160 |
| KIAA1671 | MAPK1 | 0.663 | 1.53E-155 | 1.33E-152 |
| USP34 | MAPK1 | 0.639 | 1.94E-141 | 1.11E-138 |
| MAPK1 | NUP50 | 0.638 | 6.88E-141 | 3.88E-138 |
| MIEF1 | MAPK1 | 0.626 | 6.21E-134 | 2.83E-131 |
| CREB1 | MAPK1 | 0.619 | 4.08E-130 | 1.65E-127 |
| MAPK1 | BCL2L13 | 0.613 | 5.60E-127 | 2.04E-124 |
| MAPK1 | NCKAP1 | 0.599 | 5.57E-120 | 1.61E-117 |
| STAT3 | DHX8 | 0.581 | 2.93E-111 | 6.28E-109 |
| GPATCH8 | STAT3 | 0.55 | 1.44E-97 | 1.88E-95 |
| STAT3 | KANSL1 | 0.539 | 5.79E-93 | 6.40E-91 |
| STAT3 | ASH1L | 0.516 | 4.64E-84 | 3.66E-82 |
| NBR1 | STAT3 | 0.513 | 8.39E-83 | 6.29E-81 |
| STAT3 | BECN1 | 0.512 | 1.02E-82 | 7.63E-81 |
| ANKFY1 | STAT3 | 0.511 | 2.78E-82 | 2.04E-80 |
| STAT3 | SLMAP | 0.486 | 2.46E-73 | 1.27E-71 |
| STAT3 | MFAP3 | 0.485 | 6.22E-73 | 3.15E-71 |
| STAT3 | FNDC3A | 0.483 | 1.98E-72 | 9.81E-71 |
| CDCA5 | BIRC5 | 0.909 | 0.00E+00 | 0.00E+00 |
| UBE2C | BIRC5 | 0.902 | 0.00E+00 | 0.00E+00 |
| KIF2C | BIRC5 | 0.9 | 0.00E+00 | 0.00E+00 |
| PLK1 | BIRC5 | 0.9 | 0.00E+00 | 0.00E+00 |
| BIRC5 | TPX2 | 0.896 | 0.00E+00 | 0.00E+00 |
| CCNB2 | BIRC5 | 0.893 | 0.00E+00 | 0.00E+00 |
| AURKB | BIRC5 | 0.884 | 0.00E+00 | 0.00E+00 |
| CDCA8 | BIRC5 | 0.882 | 0.00E+00 | 0.00E+00 |
| MYBL2 | BIRC5 | 0.881 | 0.00E+00 | 0.00E+00 |
| CCNA2 | BIRC5 | 0.88 | 0.00E+00 | 0.00E+00 |
| EMILIN1 | TGFB1 | 0.678 | 4.17E-165 | 4.66E-162 |
| COL6A2 | TGFB1 | 0.67 | 4.74E-160 | 4.64E-157 |
| LOXL1 | TGFB1 | 0.657 | 3.62E-152 | 2.84E-149 |
| BGN | TGFB1 | 0.64 | 1.04E-141 | 6.02E-139 |
| CLEC11A | TGFB1 | 0.639 | 3.04E-141 | 1.73E-138 |

| THY1 | TGFB1 | 0.634 | 2.03E-138 | 1.06E-135 |
| --- | --- | --- | --- | --- |
| COL6A1 | TGFB1 | 0.63 | 3.03E-136 | 1.48E-133 |
| PCOLCE | TGFB1 | 0.627 | 1.59E-134 | 7.39E-132 |
| IGFBP7 | TGFB1 | 0.627 | 1.82E-134 | 8.42E-132 |
| TGFB1 | SPI1 | 0.625 | 3.97E-133 | 1.76E-130 |
| CTSK | MMP2 | 0.902 | 0.00E+00 | 0.00E+00 |
| HTRA1 | MMP2 | 0.88 | 0.00E+00 | 0.00E+00 |
| MMP2 | TIMP2 | 0.839 | 0.00E+00 | 0.00E+00 |
| LUM | MMP2 | 0.83 | 2.915e-  311 | 5.47E-307 |
| COL1A2 | MMP2 | 0.819 | 5.49E-297 | 8.24E-293 |
| SPARC | MMP2 | 0.818 | 5.77E-295 | 8.34E-291 |
| GLT8D2 | MMP2 | 0.815 | 1.54E-291 | 2.10E-287 |
| PCOLCE | MMP2 | 0.812 | 1.87E-287 | 2.38E-283 |
| MMP2 | DCN | 0.807 | 6.32E-282 | 7.33E-278 |
| CRISPLD2 | MMP2 | 0.806 | 1.37E-279 | 1.52E-275 |
| CXCL12 | DCN | 0.829 | 1.053e-  310 | 1.96E-306 |
| TGFBR2 | CXCL12 | 0.809 | 2.39E-284 | 2.88E-280 |
| CXCL12 | ECM2 | 0.798 | 3.01E-270 | 2.87E-266 |
| CXCL12 | CCDC80 | 0.788 | 2.24E-259 | 1.77E-255 |
| SLIT3 | CXCL12 | 0.781 | 1.48E-251 | 1.01E-247 |
| LHFPL6 | CXCL12 | 0.77 | 1.66E-240 | 9.24E-237 |
| MFAP4 | CXCL12 | 0.77 | 3.54E-240 | 1.95E-236 |
| DPT | CXCL12 | 0.77 | 4.22E-240 | 2.32E-236 |
| KCTD12 | CXCL12 | 0.767 | 2.42E-237 | 1.27E-233 |
| SPARCL1 | CXCL12 | 0.765 | 3.13E-235 | 1.58E-231 |
| ENAH | KDM5B | 0.657 | 8.11E-152 | 6.31E-149 |
| ACBD3 | ENAH | 0.646 | 2.23E-145 | 1.44E-142 |
| ENAH | FBXO28 | 0.644 | 6.10E-144 | 3.77E-141 |
| WDR26 | ENAH | 0.632 | 2.38E-137 | 1.20E-134 |
| ENAH | RPS6KC1 | 0.62 | 8.91E-131 | 3.67E-128 |
| ENAH | CDC73 | 0.61 | 1.33E-125 | 4.64E-123 |
| IPO9 | ENAH | 0.607 | 1.17E-123 | 3.83E-121 |
| BROX | ENAH | 0.595 | 1.13E-117 | 3.03E-115 |
| ENAH | RAB3GAP2 | 0.59 | 1.16E-115 | 2.89E-113 |
| ENAH | INTS7 | 0.586 | 1.16E-113 | 2.70E-111 |
| RAB31 | GJA1 | 0.501 | 8.82E-79 | 5.64E-77 |
| GJA1 | PGR | 0.474 | 1.88E-69 | 8.26E-68 |
| GJA1 | OMD | 0.452 | 1.42E-62 | 4.72E-61 |
| GJA1 | LRRC17 | 0.442 | 1.07E-59 | 3.15E-58 |
| GJA1 | IL6ST | 0.435 | 1.72E-57 | 4.60E-56 |

| GJA1 | FRMD6 | 0.429 | 5.51E-56 | 1.38E-54 |
| --- | --- | --- | --- | --- |
| GJA1 | COL12A1 | 0.427 | 2.33E-55 | 5.69E-54 |
| GNG12 | GJA1 | 0.427 | 3.39E-55 | 8.23E-54 |
| GJA1 | ELOVL5 | 0.426 | 4.85E-55 | 1.17E-53 |
| GJA1 | PDGFC | 0.424 | 1.48E-54 | 3.50E-53 |
| YAP1 | BIRC2 | 0.677 | 2.10E-164 | 2.31E-161 |
| YAP1 | PICALM | 0.675 | 2.45E-163 | 2.62E-160 |
| YAP1 | QKI | 0.66 | 5.46E-154 | 4.52E-151 |
| CWF19L2 | YAP1 | 0.659 | 2.91E-153 | 2.36E-150 |
| ANO6 | YAP1 | 0.658 | 1.00E-152 | 7.99E-150 |
| SIK2 | YAP1 | 0.652 | 9.50E-149 | 6.77E-146 |
| SOCS5 | YAP1 | 0.652 | 9.96E-149 | 7.10E-146 |
| ARID5B | YAP1 | 0.65 | 7.29E-148 | 5.06E-145 |
| AMOTL1 | YAP1 | 0.648 | 2.45E-146 | 1.63E-143 |
| YAP1 | VCL | 0.64 | 5.29E-142 | 3.09E-139 |
| TNFAIP3 | ICAM1 | 0.657 | 8.07E-152 | 6.28E-149 |
| GBP1 | ICAM1 | 0.642 | 1.08E-142 | 6.44E-140 |
| SOD2 | ICAM1 | 0.631 | 9.62E-137 | 4.77E-134 |
| RELB | ICAM1 | 0.631 | 1.43E-136 | 7.08E-134 |
| BCL2A1 | ICAM1 | 0.61 | 3.05E-125 | 1.05E-122 |
| IRF1 | ICAM1 | 0.608 | 1.53E-124 | 5.15E-122 |
| RNF19B | ICAM1 | 0.604 | 3.05E-122 | 9.53E-120 |
| ICAM1 | IL32 | 0.603 | 4.66E-122 | 1.45E-119 |
| ICAM1 | BIRC3 | 0.599 | 8.90E-120 | 2.56E-117 |
| LYN | ICAM1 | 0.591 | 6.56E-116 | 1.65E-113 |

**Table S2.** Negatively correlated genes from TCSBN

| **mRNAs** | **correlated mRNAs** | **correlation** | **p-value** | **adj p-value** |
| --- | --- | --- | --- | --- |
| EGFR | GATA3 | -0.665 | 1.48E-156 | 1.32E-153 |
| EGFR | NECAB3 | -0.658 | 1.02E-152 | 8.15E-150 |
| POLR3K | EGFR | -0.652 | 9.60E-149 | 6.84E-146 |
| SMIM22 | EGFR | -0.65 | 1.64E-147 | 1.13E-144 |
| COX6C | EGFR | -0.649 | 3.47E-147 | 2.36E-144 |
| HID1 | EGFR | -0.633 | 5.62E-138 | 2.90E-135 |
| EGFR | FOXA1 | -0.63 | 7.07E-136 | 3.42E-133 |
| EGFR | COPZ1 | -0.619 | 5.32E-130 | 2.14E-127 |
| SNRNP25 | EGFR | -0.618 | 8.48E-130 | 3.39E-127 |
| KLHDC9 | EGFR | -0.617 | 4.59E-129 | 1.79E-126 |
| NDUFA13 | MAPK1 | -0.588 | 2.45E-114 | 5.85E-112 |

| BLOC1S1 | MAPK1 | -0.578 | 7.85E-110 | 1.60E-107 |
| --- | --- | --- | --- | --- |
| EDF1 | MAPK1 | -0.575 | 1.18E-108 | 2.31E-106 |
| ZNHIT1 | MAPK1 | -0.569 | 5.73E-106 | 1.02E-103 |
| MRPL41 | MAPK1 | -0.569 | 1.40E-105 | 2.45E-103 |
| MAPK1 | SIRT6 | -0.567 | 8.90E-105 | 1.52E-102 |
| MAPK1 | ATP5F1D | -0.558 | 4.27E-101 | 6.37E-99 |
| ELOB | MAPK1 | -0.557 | 2.42E-100 | 3.51E-98 |
| METTL26 | MAPK1 | -0.555 | 9.50E-100 | 1.35E-97 |
| FASTK | MAPK1 | -0.55 | 2.17E-97 | 2.84E-95 |
| ATP5MD | STAT3 | -0.451 | 2.39E-62 | 7.83E-61 |
| STAT3 | ATP5F1E | -0.443 | 8.79E-60 | 2.59E-58 |
| MT-CO2 | STAT3 | -0.443 | 9.02E-60 | 2.66E-58 |
| ATP5ME | STAT3 | -0.436 | 5.55E-58 | 1.52E-56 |
| STAT3 | COX4I1 | -0.426 | 3.68E-55 | 8.91E-54 |
| STAT3 | FAM96B | -0.426 | 3.91E-55 | 9.46E-54 |
| STAT3 | ATP5MPL | -0.415 | 3.89E-52 | 8.26E-51 |
| BANF1 | STAT3 | -0.414 | 7.02E-52 | 1.48E-50 |
| STAT3 | COX5B | -0.412 | 2.36E-51 | 4.84E-50 |
| BCAP31 | STAT3 | -0.41 | 7.48E-51 | 1.50E-49 |
| BIRC5 | CYBRD1 | -0.705 | 1.92E-184 | 3.44E-181 |
| ZBTB4 | BIRC5 | -0.696 | 7.61E-178 | 1.17E-174 |
| CRY2 | BIRC5 | -0.694 | 1.81E-176 | 2.69E-173 |
| TNS2 | BIRC5 | -0.691 | 3.82E-174 | 5.35E-171 |
| BIRC5 | CALCOCO1 | -0.671 | 1.98E-160 | 1.96E-157 |
| PDGFD | BIRC5 | -0.651 | 1.76E-148 | 1.24E-145 |
| NOSTRIN | BIRC5 | -0.634 | 1.79E-138 | 9.38E-136 |
| SPARCL1 | BIRC5 | -0.617 | 7.50E-129 | 2.91E-126 |
| BIRC5 | NTN4 | -0.611 | 3.60E-126 | 1.28E-123 |
| OGN | BIRC5 | -0.611 | 4.05E-126 | 1.44E-123 |
| RMND5A | TGFB1 | -0.447 | 4.07E-61 | 1.27E-59 |
| TOMM70 | TGFB1 | -0.44 | 6.00E-59 | 1.71E-57 |
| TGFB1 | TXLNG | -0.384 | 3.13E-44 | 4.68E-43 |
| LRPPRC | TGFB1 | -0.382 | 8.07E-44 | 1.19E-42 |
| UBA2 | TGFB1 | -0.38 | 2.78E-43 | 3.98E-42 |
| TGFB1 | RB1CC1 | -0.38 | 2.79E-43 | 4.00E-42 |
| TGFB1 | MCCC1 | -0.38 | 3.06E-43 | 4.37E-42 |
| OPA1 | TGFB1 | -0.379 | 6.39E-43 | 9.00E-42 |
| TFAM | TGFB1 | -0.376 | 1.95E-42 | 2.69E-41 |
| TGFB1 | ZFR | -0.376 | 2.14E-42 | 2.95E-41 |
| MRPS33 | MMP2 | -0.426 | 5.52E-55 | 1.33E-53 |

| ENOPH1 | MMP2 | -0.414 | 1.14E-51 | 2.36E-50 |
| --- | --- | --- | --- | --- |
| KRTCAP3 | MMP2 | -0.409 | 2.27E-50 | 4.46E-49 |
| HSPD1 | MMP2 | -0.404 | 4.68E-49 | 8.68E-48 |
| PAICS | MMP2 | -0.398 | 9.78E-48 | 1.71E-46 |
| CHMP4C | MMP2 | -0.394 | 1.34E-46 | 2.23E-45 |
| MMP2 | MTIF2 | -0.393 | 2.41E-46 | 3.97E-45 |
| PRELID3B | MMP2 | -0.392 | 2.90E-46 | 4.76E-45 |
| WRNIP1 | MMP2 | -0.387 | 7.90E-45 | 1.22E-43 |
| PDHX | MMP2 | -0.386 | 8.27E-45 | 1.27E-43 |
| CXCL12 | E2F1 | -0.56 | 1.32E-101 | 2.01E-99 |
| UBE2S | CXCL12 | -0.555 | 9.55E-100 | 1.36E-97 |
| CDT1 | CXCL12 | -0.542 | 4.00E-94 | 4.62E-92 |
| UBE2C | CXCL12 | -0.536 | 7.14E-92 | 7.58E-90 |
| TPI1 | CXCL12 | -0.534 | 3.71E-91 | 3.83E-89 |
| CXCL12 | UBE2T | -0.534 | 6.53E-91 | 6.68E-89 |
| GINS2 | CXCL12 | -0.532 | 4.04E-90 | 4.01E-88 |
| CXCL12 | RNASEH2A | -0.528 | 8.65E-89 | 8.16E-87 |
| KIFC1 | CXCL12 | -0.527 | 1.98E-88 | 1.85E-86 |
| CCNB1 | CXCL12 | -0.526 | 9.63E-88 | 8.74E-86 |
| ENAH | CST3 | -0.463 | 6.26E-66 | 2.39E-64 |
| ENAH | YPEL3 | -0.45 | 4.78E-62 | 1.55E-60 |
| ENAH | FCGRT | -0.428 | 1.54E-55 | 3.79E-54 |
| ENAH | PFDN5 | -0.408 | 2.69E-50 | 5.28E-49 |
| ENAH | UROD | -0.403 | 6.38E-49 | 1.18E-47 |
| ENAH | POLR2E | -0.401 | 2.46E-48 | 4.42E-47 |
| ENAH | SCRN2 | -0.396 | 3.83E-47 | 6.53E-46 |
| ENAH | C12orf10 | -0.395 | 6.18E-47 | 1.04E-45 |
| ENAH | SAT2 | -0.393 | 2.17E-46 | 3.58E-45 |
| C1orf123 | ENAH | -0.383 | 4.36E-44 | 6.48E-43 |
| C6orf136 | GJA1 | -0.383 | 6.05E-44 | 8.93E-43 |
| CDT1 | GJA1 | -0.374 | 7.15E-42 | 9.61E-41 |
| GJA1 | EMC8 | -0.362 | 4.80E-39 | 5.67E-38 |
| GJA1 | FAM136A | -0.361 | 6.31E-39 | 7.40E-38 |
| GJA1 | NUTF2 | -0.356 | 6.59E-38 | 7.38E-37 |
| GJA1 | SNRPA | -0.355 | 1.42E-37 | 1.56E-36 |
| GJA1 | MRPL2 | -0.349 | 2.05E-36 | 2.13E-35 |
| FAM96B | GJA1 | -0.349 | 2.36E-36 | 2.45E-35 |
| GJA1 | KLHDC3 | -0.345 | 2.23E-35 | 2.22E-34 |
| MVD | GJA1 | -0.344 | 2.37E-35 | 2.35E-34 |
| YAP1 | TIMM17B | -0.638 | 1.34E-140 | 7.48E-138 |

| COX17 | YAP1 | -0.629 | 8.70E-136 | 4.20E-133 |
| --- | --- | --- | --- | --- |
| YAP1 | UBE2M | -0.628 | 3.82E-135 | 1.81E-132 |
| YAP1 | ATP6V0B | -0.61 | 2.62E-125 | 9.05E-123 |
| YAP1 | COX6A1 | -0.606 | 1.78E-123 | 5.78E-121 |
| ANAPC11 | YAP1 | -0.603 | 6.81E-122 | 2.10E-119 |
| YAP1 | DYNLRB1 | -0.601 | 5.09E-121 | 1.52E-118 |
| NME1 | YAP1 | -0.6 | 1.52E-120 | 4.49E-118 |
| YAP1 | PAFAH1B3 | -0.596 | 2.63E-118 | 7.18E-116 |
| GDI1 | YAP1 | -0.59 | 1.17E-115 | 2.91E-113 |
| CDKN2AIPNL | ICAM1 | -0.422 | 7.85E-54 | 1.79E-52 |
| REEP5 | ICAM1 | -0.411 | 4.90E-51 | 9.92E-50 |
| IRX5 | ICAM1 | -0.408 | 3.07E-50 | 6.00E-49 |
| CETN3 | ICAM1 | -0.387 | 6.41E-45 | 9.89E-44 |
| NECAB3 | ICAM1 | -0.38 | 3.10E-43 | 4.43E-42 |
| PLA2G12A | ICAM1 | -0.379 | 4.42E-43 | 6.27E-42 |
| COX6C | ICAM1 | -0.375 | 3.43E-42 | 4.68E-41 |
| HID1 | ICAM1 | -0.375 | 4.23E-42 | 5.75E-41 |
| GAMT | ICAM1 | -0.369 | 1.01E-40 | 1.28E-39 |
| ICAM1 | PEBP1 | -0.368 | 1.45E-40 | 1.83E-39 |


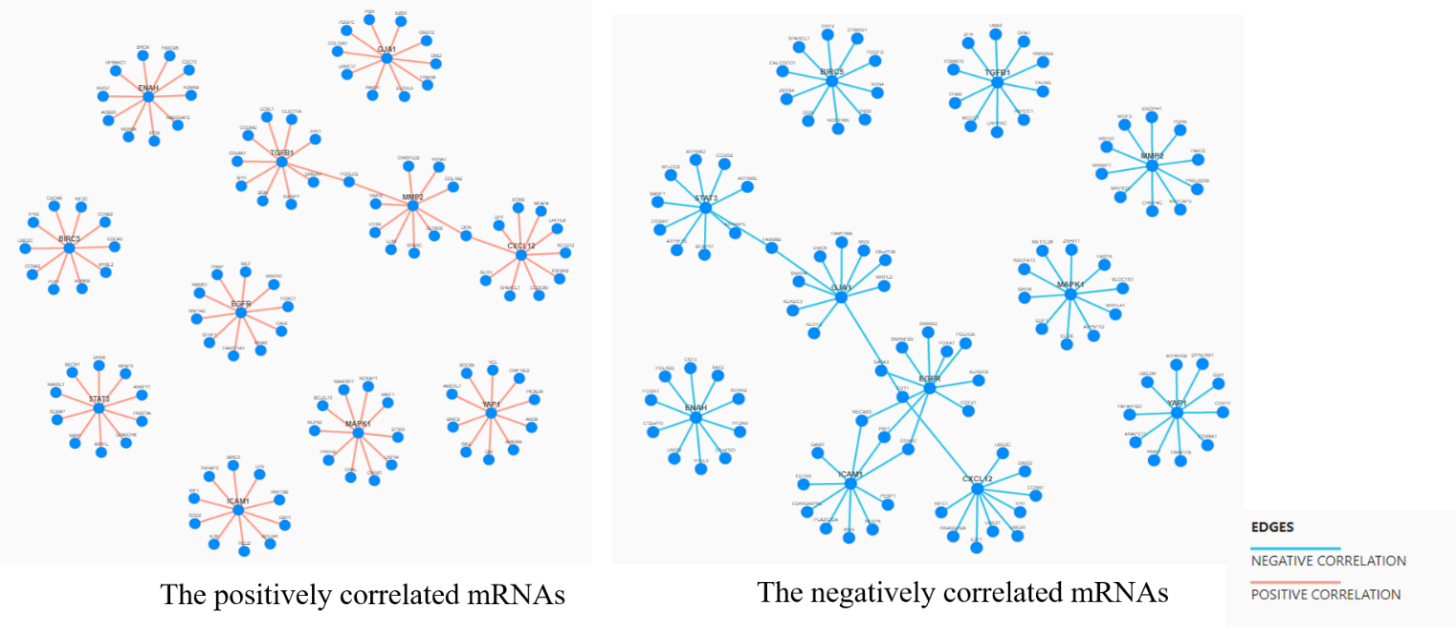


**Genes**

**Fig S3.** BC-specific backbone gene co-expression

**Table S3**. CheA3 TF analysis

| **Rank** | **TF** | **Score** |
| --- | --- | --- |
| 1 | CSRNP1 | 43.5 |
| 2 | BHLHE40 | 45 |
| 3 | IRF3 | 45.2 |
| 4 | TLX1 | 57.67 |
| 5 | ZNF384 | 58.6 |
| 6 | ESR1 | 81.33 |
| 7 | NFATC2 | 84.33 |
| 8 | ETS1 | 156.7 |
| 9 | IRF4 | 94.2 |
| 10 | RXRA | 97.17 |
| 11 | RUNX1 | 98.4 |
| 12 | STAT5A | 101 |
| 13 | MYB | 101.7 |
| 14 | SP1 | 236.6 |

**Table S4.** Different mutations occurring at different chromosomal positions for circos plot

| **Data** | **FD** | **MS** | **SP** |
| --- | --- | --- | --- |
| BIRC5 | 142 | 166 | 1 |
| STAT3_1 | 0 | 410 | 0 |
| STAT3_2 | 0 | 384 | 0 |
| STAT3_3 | 0 | 724 | 0 |
| STAT3_4 | 0 | 122 | 0 |
| STAT3_5 | 0 | 423 | 0 |
| MAPK1 | 0 | 81 | 0 |
| TGFB1 | 197 | 296 | 0 |
| MMP2_1 | 0 | 372 | 0 |
| MMP2_2 | 0 | 262 | 0 |
| EGFR_1 | 0 | 114 | 0 |
| EGFR_2 | 0 | 273 | 0 |
| EGFR_3 | 0 | 105 | 0 |
| EGFR_4 | 0 | 314 | 0 |
| ENAH_1 | 195 | 232 | 58 |
| ENAH_2 | 0 | 200 | 0 |
| ENAH_3 | 0 | 423 | 0 |
| ENAH_4 | 0 | 390 | 0 |
| ENAH_5 | 0 | 98 | 0 |
| GJA1_1 | 0 | 317 | 0 |
| GJA1_2 | 0 | 183 | 0 |
| GJA1_3 | 0 | 319 | 0 |
| GJA1_4 | 0 | 127 | 0 |
| YAP1 | 0 | 472 | 0 |
| ICAM1_1 | 0 | 218 | 0 |
| ICAM1_2 | 0 | 201 | 0 |


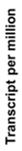

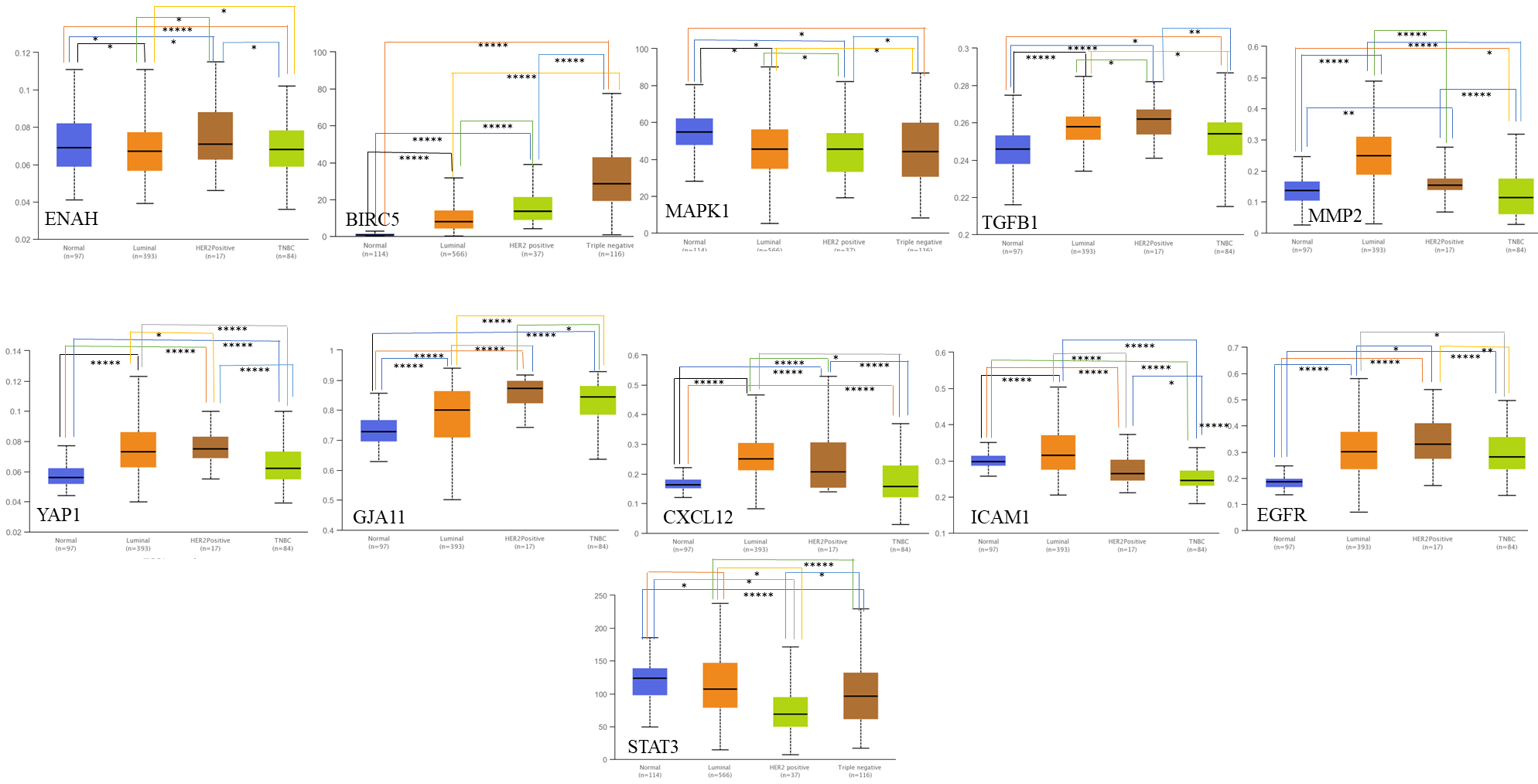


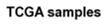


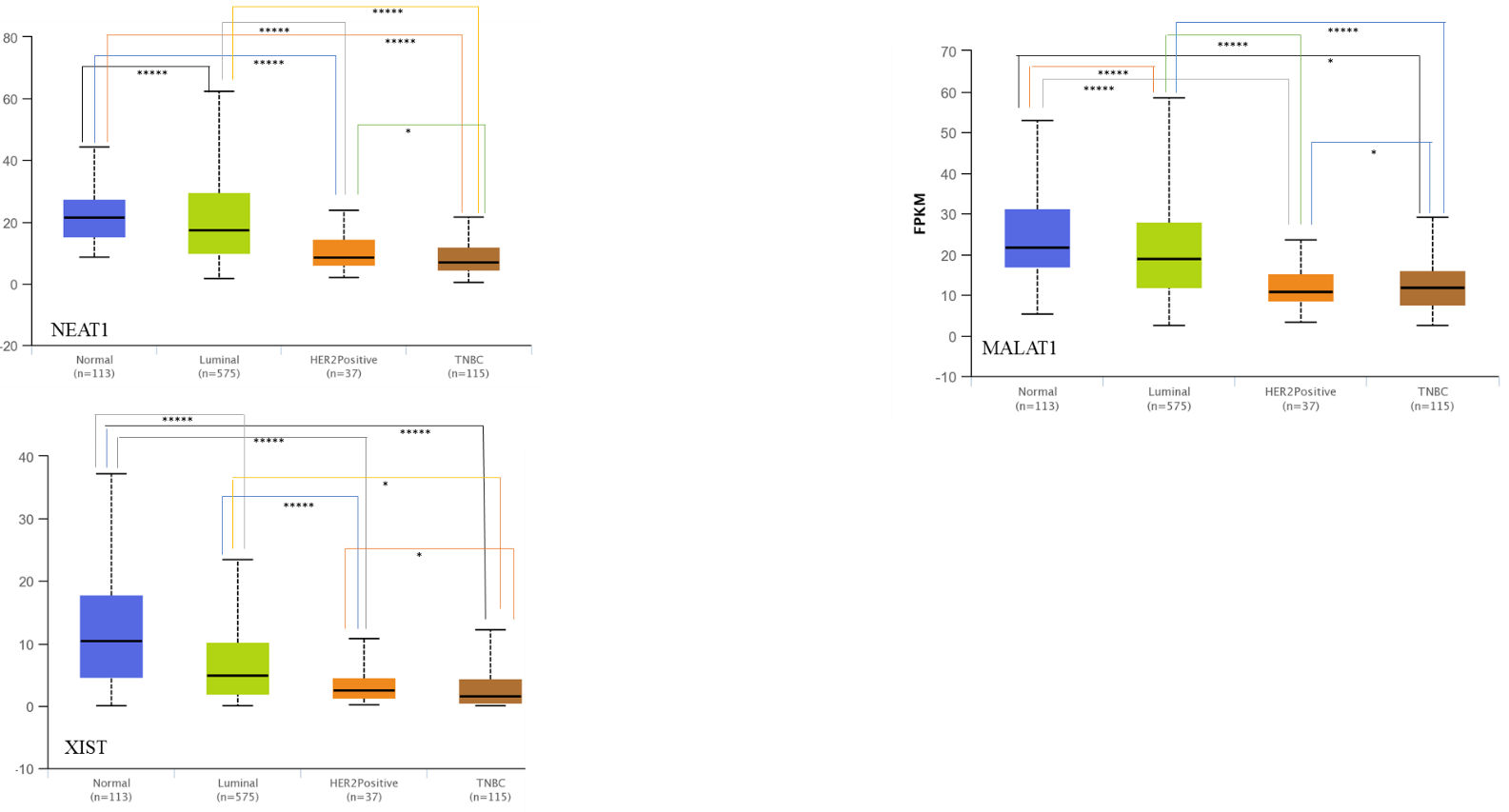

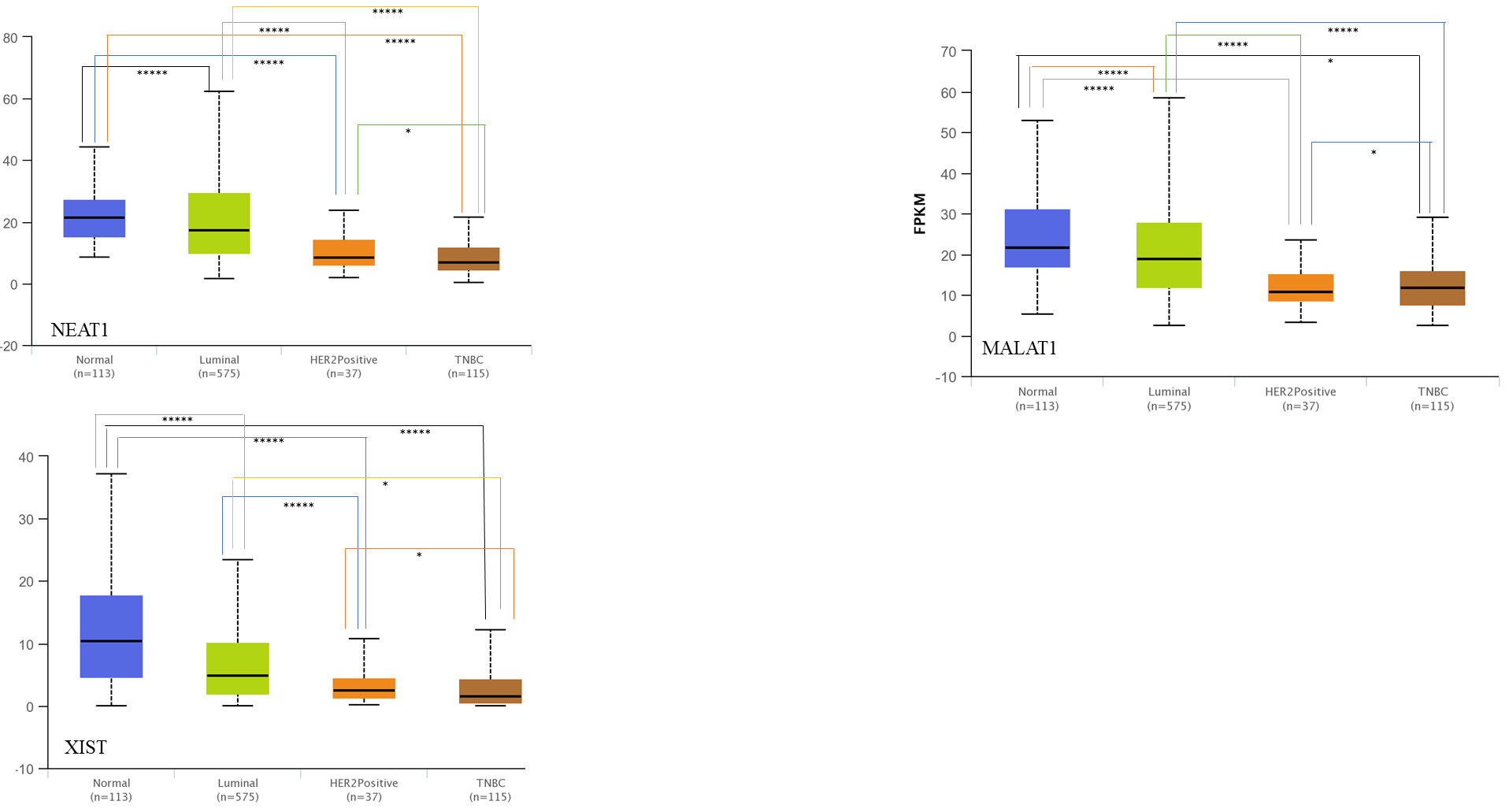

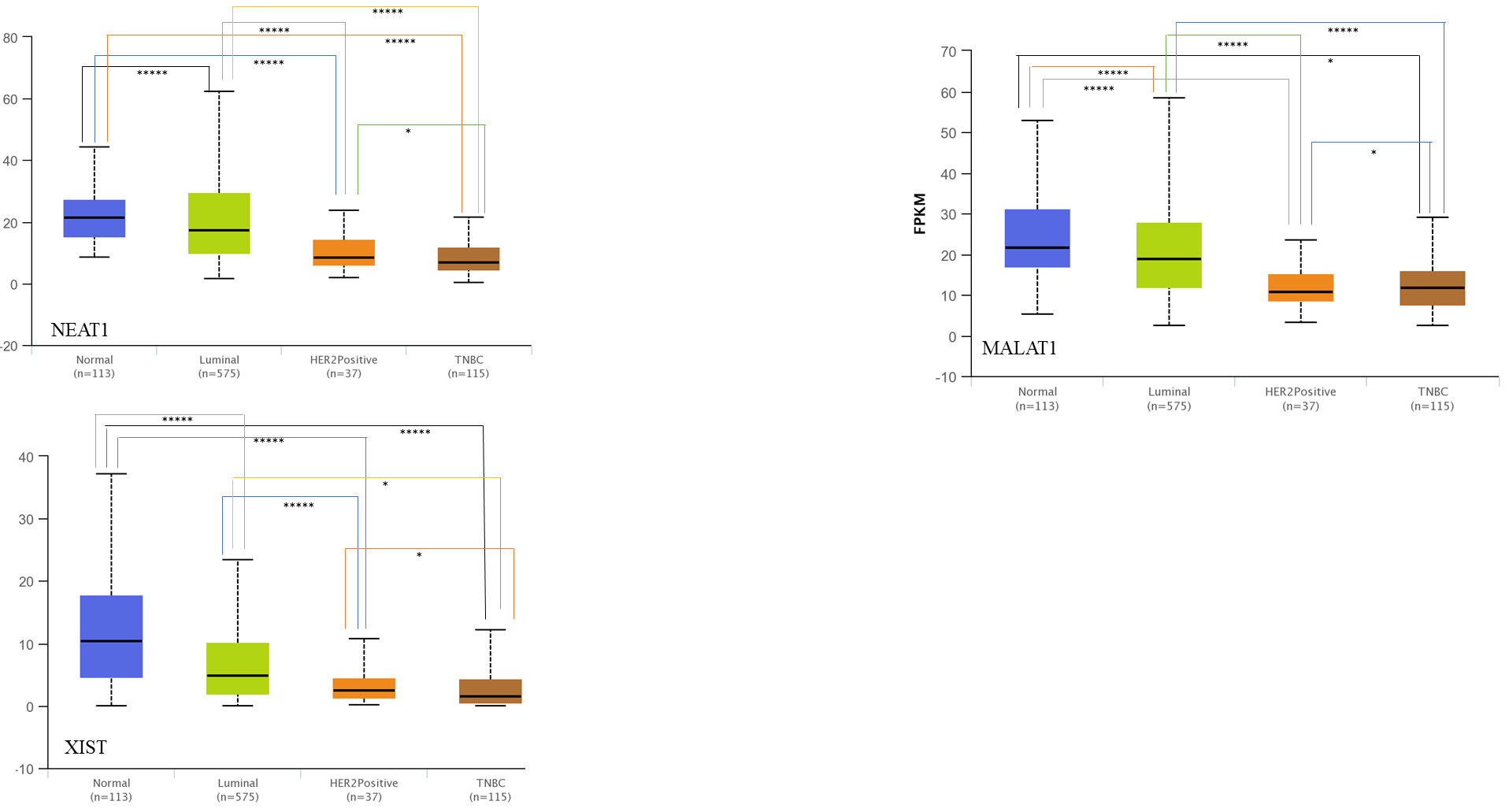
**Fig S4.** The subtype-based mRNA expression based on TCGA dataset

**Fig S5.** The subtype-based lncRNA expression based on TCGA dataset


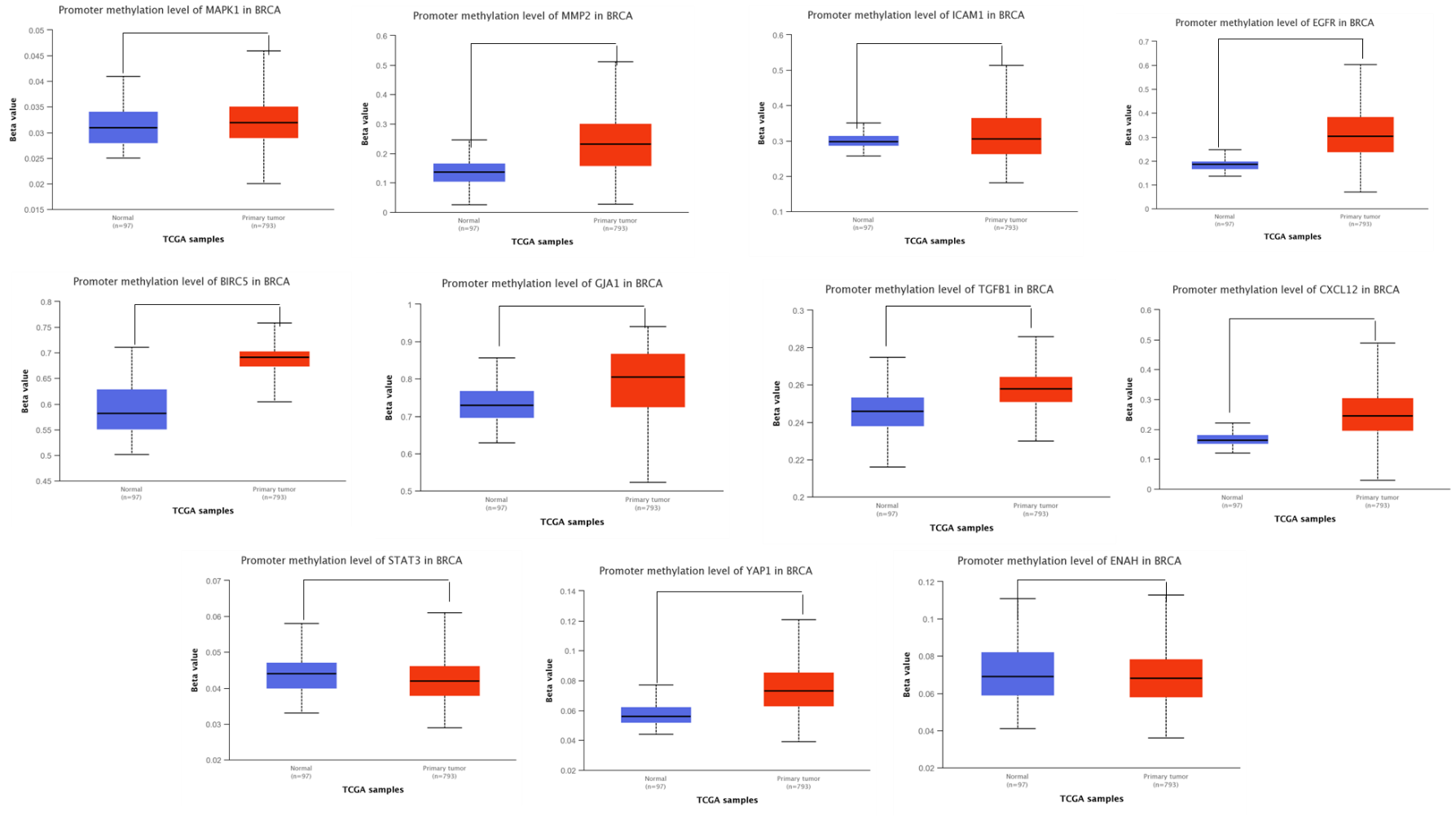


**Fig S6.** The promoter-based methylation of lncRNA expression based on TCGA dataset
